# Characterization of pyridylpiperazine-based efflux pump inhibitors for *Acinetobacter baumannii*

**DOI:** 10.1093/jacamr/dlad112

**Published:** 2023-10-24

**Authors:** Juan-Carlos Jiménez-Castellanos, Elizabeth Pradel, Nina Compagne, Anais Vieira Da Cruz, Marion Flipo, Ruben C Hartkoorn

**Affiliations:** Université de Lille, CNRS, INSERM, CHU Lille, Institut Pasteur de Lille, U1019, UMR 9017, CIIL, Center for Infection and Immunity of Lille, F-59000 Lille, France; Université de Lille, CNRS, INSERM, CHU Lille, Institut Pasteur de Lille, U1019, UMR 9017, CIIL, Center for Infection and Immunity of Lille, F-59000 Lille, France; Université de Lille, INSERM, Institut Pasteur de Lille, U1177, Drugs and Molecules for Living Systems, F-59000 Lille, France; Université de Lille, INSERM, Institut Pasteur de Lille, U1177, Drugs and Molecules for Living Systems, F-59000 Lille, France; Université de Lille, INSERM, Institut Pasteur de Lille, U1177, Drugs and Molecules for Living Systems, F-59000 Lille, France; Université de Lille, CNRS, INSERM, CHU Lille, Institut Pasteur de Lille, U1019, UMR 9017, CIIL, Center for Infection and Immunity of Lille, F-59000 Lille, France

## Abstract

**Objectives:**

In *Acinetobacter baumannii*, multidrug efflux pumps belonging to the resistance-nodulation-division (RND) superfamily result in decreased antibiotic susceptibility. Improving the activity of current antibiotics via efflux pump inhibitors (EPIs) represents an attractive alternative approach to control this bacterium. Pyridylpiperazines (PyrPips) are a new class of EPIs that can effectively inhibit the *Escherichia coli* RND efflux pump AcrAB-TolC and boost the activity of several antibiotics. Here we have evaluated and characterized whether the PyrPip chemical family is also able to boost antibiotic activity through inhibition of the RND efflux pumps in *A. baumannii*.

**Methods:**

Comparative structural modelling and docking, structure-activity relationship studies alongside molecular genetic approaches were deployed to improve, characterize and validate PyrPips’ target.

**Results:**

We showed that two enhanced PyrPip EPIs are capable of rescuing the activity of different classes of antibiotics in *A. baumannii*. By expressing *A. baumannii* main efflux pumps (AdeB, AdeG and AdeJ) individually in *E. coli* recombinant strains, we could gain further insights about the EPIs’ capacity to act upon each pump. Finally, we showed that PyrPip EPIs are mostly acting through AdeJ inhibition via interactions with two key charged residues, namely E959 and E963.

**Conclusions:**

Our work demonstrates that PyrPip EPIs are capable of inhibiting RND efflux pumps of *A. baumannii,* and thus may present a promising chemical scaffold for further development.

## Introduction

*Acinetobacter baumannii* is a critical emerging Gram-negative pathogen, now considered by the WHO and CDC as a health priority for research and development (R&D).^1^*A. baumannii* exploits diverse mechanisms to elude the action of antibiotics, including the use of efflux pumps to extrude a myriad of xenobiotics from within the bacteria.^2,3^ In *A. baumannii,* like in many other Gram-negative bacteria, efflux pumps belonging to the resistance-nodulation-division (RND) superfamily play a clinically relevant role in antibiotic resistance.^4^ Although at least nine have been described for *A. baumannii*, the AdeABC, AdeFGH and AdeIJK tripartite systems have been primarily linked to loss of antibiotic susceptibility.^4,5^

The fundamental role of RND efflux pumps in reducing antibiotic susceptibility in Gram-negative bacteria has prompted great interest in developing efflux pump inhibitors (EPIs) over the last 15 years. Primary examples of such discovered and characterized EPIs targeting Gram-negative RND pumps include the peptidomimetic PAβN,^6–8^ pyranopyridines of the MBX series^9–11^ and the pyridopyrimidine D13-9001.^12^ In addition to these EPIs, we recently described and characterized a new class of EPIs, known as pyridylpiperazines (PyrPips),^13,14^ that boost antibiotic activity in *Escherichia coli* by binding a novel allosteric pocket in the transmembrane region of AcrB and inhibiting the activity of the AcrAB-TolC efflux pump.

*A. baumannii* is known to have low membrane permeability, driven by a combination of porins and efficient efflux pumps that hamper antibiotic activity.^15^ In *A. baumannii,* the main efflux pumps (i.e. AdeB, AdeG and AdeJ) have an overall low degree of amino acid sequence conservation when compared with AcrB of *E. coli* (49%, 40% and 57%, respectively); however, the residues around our previously described PyrPip inhibitor-binding site are highly conserved. In this work, we sought to investigate whether PyrPip could act as an EPI of *A. baumannii* RND pumps. Validation and characterization of PyrPips’ target was achieved by deploying comparative structural modelling and docking, structure-activity relationship studies and molecular genetics approaches. We show that two enhanced EPIs are capable of rescuing the activity of different antibiotic classes in *A. baumannii*. We report that although our enhanced EPIs act primarily on AdeJ and to a lesser extent on AdeG and AdeB, further efforts are required to improve our knowledge behind the biology of *A. baumannii* efflux pumps, leading to more potent PyrPip EPIs to help combat antimicrobial resistance mediated by efflux pumps.

## Materials and methods

### Chemical synthesis of pyridylpiperazines

The chemical synthesis can be found in the Supplementary Material (available as Supplementary data at *JAC-AMR* Online).

### Bacterial strains and reagents

Bacterial strains, plasmids and primers used are listed in Tables S1 and S2. Bacteria were regularly cultured on LB broth (BD, DIFCO) or CAMHB at 37°C. When necessary, kanamycin or gentamicin were added to the cultures at a final concentration of 50 mg/L or 10 mg/L, respectively. Antibiotics and PAβN were purchased from Sigma Aldrich, Carbosynth Limited, Fisher Scientific and Euromedex.

### MICs

The MICs of antibiotics against *A. baumannii* were determined according to the CLSI guidelines^16^ with slight modifications. Briefly, overnight bacterial cultures were diluted to OD_600_ = 0.001 in CAMHB. Where appropriate the bacterial culture was spiked with the EPIs of interest at indicated concentrations. These bacterial suspensions were transferred to a 96-well flat-bottomed microtitre plate (Falcon, France), and a gradient of antibiotics of interest was added by serial dilutions. Plates were incubated at 37 °C for up to 20 h, and bacterial viability was evaluated using the resazurin reduction assay and measured by fluorescence (POLARstar Omega, BMG Labtech: Ex: 530 nm, Em: 590 nm). MICs were defined as the concentration that prevented 90% of resazurin turnover compared with the non-treated bacteria.

### Heterologous expression of the Ade efflux pumps in E. coli ΔacrAB

*E. coli* Δ*acrAB*, lacking its major RND efflux pump, was generated as previously described.^17^ To express the *A. baumannii* RND pumps from the *E. coli* Δ*acrAB* chromosome, each pump operon was first cloned in an oriR6K suicide plasmid bearing the *E. coli acrR* gene and PacrA promoter. The recombinant plasmids were constructed using an NEBuilder assembly kit and the Q5-HF polymerase (NEB, France). The *acrR-PacrA* DNA fragment was PCR amplified from *E. coli* Δ*acrAB* genomic DNA. The *adeABC* and *adeIJK* operons were PCR amplified from the genome of *A. baumannii* AB5075, and *adeFGH* from *A. baumannii* ATCC17978. Each assembly mix was transformed into *E. coli* UCC/*pir+* (Lucigen, UK) by electroporation, and kanamycin-resistant clones were selected. Correct plasmid constructs were verified by Sanger sequencing. The generated plasmids were designated pJC1 (*PacrA-adeIJK*), pJC2 (*PacrA-adeABC*) and pJC3 (*PacrA-adeFGH*), and introduced into *E. coli* Δ*acrAB* by electroporation, selecting for kanamycin resistance. Correct plasmid integration at the *acrR* locus was assessed by PCR. To avoid plasmid excision in the absence of kanamycin, we then used Red-recombineering to exchange the *oriR6K-KmR* region with a gentamicin cassette and thus stabilize each chromosomal construct.

### Generation of adeJ efflux pump deletions in A. baumannii

To delete *adeJ* in *A. baumannii* ATCC17978, an allelic exchange strategy based on the pMo130 suicide vector was used as described by Amin *et al.*^18^ Briefly, 0.8 kb DNA fragments located up/downstream of the targeted gene were PCR amplified and cloned into pMo130 using an NEBbuilder assembly kit (NEB). Following confirmation of the correct plasmid assembly by Sanger sequencing, the suicide plasmid was transformed into *A. baumannii* ATCC17978 by electroporation. Single crossover events were selected on kanamycin; upon growth in LB + sucrose 15%, double-crossover kanamycin-sensitive strains were screened in which *adeJ* knockout was assessed by PCR. Subsequent WGS (microbesNG, Birmingham, UK) confirmed the anticipated genome sequence.

### Generation of A. baumannii adeJ point mutants

Point mutations in *adeJ* were introduced into the *A. baumannii* ATCC17978 chromosome using the allelic exchange method described previously^18^ and above. Briefly, a ∼1 kb DNA fragment centred on the *adeJ* targeted nucleotide was cloned into pCR-BluntII-TOPO (Fisher Scientific). The resulting plasmid was then used as a template for site-directed mutagenesis with mutagenic oligonucleotide primers (Table S2) and Q5-polymerase (NEB). DNA fragments confirmed to carry the desired *adeJ* mutations were then subcloned into pMo130, and ATCC17978 was electrotransformed with the resulting plasmid. The correct allelic exchange was assessed by sequencing a PCR amplicon of the targeted region, followed by WGS (microbesNG, Birmingham, UK).

### Quantitative RT-PCR

Bacterial cultures (OD_600_ ∼0.05) were incubated at 37°C with shaking (180 rpm) until the OD_600_ had reached 0.6–0.8. Bacterial RNA was then stabilized using RNAprotect Bacteria Reagent (Qiagen, France) according to the manufacturer’s instructions, and then disrupted with 200 µL lysozyme (20 mg/mL in TE buffer (10mM TriS-HCl (pH 8.0), 1 mM EDTA), 10 min), followed by addition of 700 μL RLT buffer (Qiagen) containing 150 mM β-mercaptoethanol. Cells were further disrupted by bead-beating in lysing matrix B tubes (MP Biochemicals) using a FastPrep-24 (MP Biomedicals) and RNA was purified using a Qiagen RNeasy RNA purification kit according to the manufacturer’s instructions. Contaminating genomic DNA was digested using TURBO DNA-free^™^ Kit (Ambion), and DNA-free RNA was then reverse transcribed to cDNA using LunaSript^™^ SuperMix kit (NEB) and random hexamers. Quantitative RT-PCR entailed using the KAPA SYBR FAST Mix (Sigma-Aldrich, France) and 200 nM of each primer (Table S2), and performed using a LightCycler^™^ 480 (Roche). Samples were run as four biological replicates with three technical repeats each from a separate RNA purification batch of cells. Relative RNA concentrations were calculated using the 2^(−ΔCt) method.^19^

### A. baumannii efflux pump model building and molecular docking

In the absence of an Ade-PyrPip co-structure, potential interactions between the EPIs and the *A. baumannii* RND efflux pumps were investigated using structural modelling by satisfaction of spatial restraints^20^ and molecular docking.^21^ The L-protomer of the BDM88855-bound AcrB (pdb: 7OUK)^13^ was used as a homologue protein template (apo-state). AdeB, G and J were modelled onto this template using python scripts^22^ for the MODELLER program,^23^ giving five output models with the lowest DOPE and SCORE levels (both underlying the lowest energy barrier required for conformational stability). The top three models (based on scores) were subjected to geometric validation using MolProbity^24^ and the best-fit model (versus AcrB L protomer) was used for docking studies with AutoDock using Vina^21^ [exhaustiveness of 4096, grid dimensions 50 Å × 50 Å × 50Å spanning transmembrane domains (TMs) 4, 5, 10 and 11]. Compounds were considered flexible during docking (number of rotatable bonds in the range 2 to 4). The top eight docking poses were kept to be used in structure-activity relationship (SAR) analysis.

## Results

### The PyrPip binding pocket is conserved in A. baumannii RND pumps

**Figure 1.**
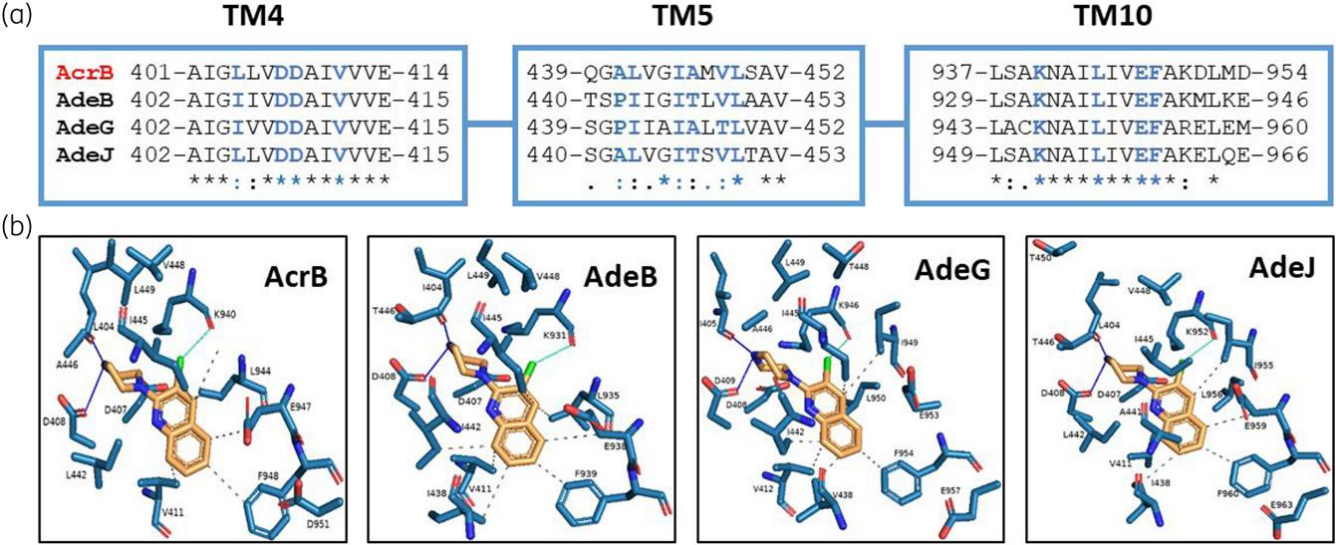
The PyrPip binding pocket in *A. baumannii* AdeB, G and J compared with that of AcrB in *E. coli*. (a) protein sequence alignment of the PyrPip binding pocket region [transmembrane domains (TMs) 4, 5 and 10] in AcrB, AdeB, AdeG and AdeJ. Residues that interact with PyrPip in AcrB (in blue) are largely conserved (*). (b) *In silico* docking of BDM88855 into the PyrPip binding pocket modelled for AdeB, AdeG and AdeJ [modelling based on the Apo structure of the L-protomer of AcrB:BDM88855 (pdb: 7OUK)]. The structural models showed no evidence of steric hindrance and highlighted the predicted conservation of PyrPip binding interactions with the Ade efflux pumps. Dotted lines: hydrophobic interactions; green lines: halogen interactions; blue lines: polar interactions.

In *A. baumannii,* three major RND-type efflux pumps are described to impact antibiotic activity, namely AdeABC, AdeFGH and AdeIJK. The inner membrane components of these tripartite pumps share moderate overall amino acid similarities when compared with AcrB of *E. coli*; however, the region of the identified PyrPip binding pocket remains highly conserved (Figure 1). The *E. coli* AcrB-PyrPip co-structure showed that PyrPip binding was associated with significant changes to the TM region,^13^ thus similar structural changes could be expected in the Ade proteins. Although the apo-structures of AdeB and AdeJ have been recently solved,^25,26^ these structures could not be used for chemical docking as they do not account for the likely required TM movement. Therefore as an initial approach to evaluate whether AdeB, AdeG and AdeJ could accommodate PyrPip EPIs, structural models of each Ade pump were generated based on the published AcrB L-protomer-BDM88885 co-structure (apo structure).^13^ Molecular docking was then used to evaluate the binding poses of the BDM88855 EPI in each of these models. The three models confirmed a high level of three-dimensional structural identity with AcrB, with only a few amino acid substitutions (Figure 1) and no evidence of obvious steric hindrance or loss of essential binding interactions. Together, the analysis suggested that PyrPips could inhibit these pumps in *A. baumannii*.

### Screening of PyrPips as EPIs in A. baumannii

Evaluation of the original PyrPip EPI hit (BDM73185) on *A. baumannii* ATCC17978 found it to be a mild booster (2–4-fold) of chloramphenicol, triclosan and linezolid antibiotic activity. Medicinal chemistry optimization of BDM73185 to more potent PyrPip analogues against *E. coli* AcrB^13^ provided an extended PyrPip chemical library (>200 compounds) that was also evaluated in *A. baumannii*. This library was assessed in dose–response against *A. baumannii* in the presence of sub-MIC concentrations of efflux pump substrate antibiotics (chloramphenicol: 50 mg/L; fusidic acid: 50 mg/L; novobiocin: 5 mg/L; erythromycin: 2.5 mg/L; azithromycin: 0.15 mg/L; and ciprofloxacin: 0.2 mg/L). Globally, this phenotypic screen clearly showed a disconnect between PyrPip potency observed in *E. coli* compared with that observed in *A. baumannii.* Overall, far fewer PyrPips were found to potentiate antibiotic activity, additionally encompassed by a lower potency. This finding suggested that despite no obvious difference in the PyrPip binding pocket of the Ade pumps, the structure-activity relationships governing the boosting of antibiotic activity were different to those established for *E. coli* AcrB.

Despite the overall inferior activity of PyrPips in *A. baumannii*, a total of 97 compounds were identified to potentiate the activity of at least one antibiotic by at least one dilution (2-fold), whereas 33 had activity to more than one antibiotic by at least two dilutions (4-fold). In general, we observed a high frequency of antibiotic boosting for fusidic acid, novobiocin and erythromycin, but to a lesser extent for azithromycin and ciprofloxacin.

The chemical structure of active PyrPips in *A. baumannii* included four compounds with a quinoline core (compounds 1–4 in Table 1) and five with a pyridine core (compounds 6–10). The three most potent EPIs from the quinoline series were close analogues with meta- (compound 1), ortho- (compound 2) and para- (compound 3) benzylamine at position 6 of the quinoline ring (Table 1). A butanediamine substituent (compound 4) also allowed for boosting of antibiotic activity, though this compound was less potent. Regarding the pyridine series, three compounds (compounds 6–8) bearing an ester linker in position 5 of the pyridine ring were able to boost fusidic acid and novobiocin, though the most potent ester (compound 8) also showed intrinsic antibacterial activity of 62 µM. The two most potent boosters in this series were analogues bearing an ethynylphenyl moiety with either an ethylamine chain in the meta- (compound 9) or para- (compound 10) position. The initial chemical library did not contain the ortho-substituted ethylamine analogue, and so it was synthesized (compound 11), and found to be 3- to 6-fold more potent at boosting novobiocin activity (EC_90_ = 10 µM) than analogues 9 (EC_90_ = 31 µM) and 10 (EC_90_ = 62 µM).

**Table 1.** Structure and activity of the PyrPip EPIs with a quinoline core (compounds 1–5) or pyridine core (compounds 6–10), identified through screening to boost the activity of at least two antibiotics by 4-fold in *A. baumannii*^a^

Compound	Core structure	R-group	Salt	EPI potency (µM) for antibiotics boosting (EC_90_)						
				Alone	CHL	Fus	Nov	ERY	AZM	CIP
BDM88855.HCl	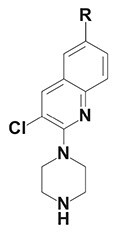	H	HCl	>125	125	125	>125	>125	>125	>125
BDM91531 1		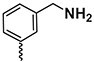	HCl	125	**15.6**	**<15**.**6**	**31.25**	**31.25**	125	125
2		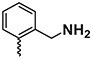	HCl	>125	125	**<15**.**6**	**<15.6**	**62.5**	125	125
3		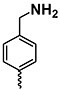	HCl	125	62.5	**<15**.**6**	**31.25**	62.5	125	>125
4		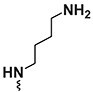	HCl	>125	125	**31**.**25**	**31.25**	125	>125	>125
5		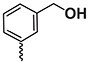	HCl	>125		>125				
BDM73185	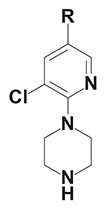	−CF_3_		>250	250	>250	>250	>250	>250	>250
6		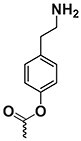	HCl	>250	>250	**125**	**125**	>250	>250	>250
7		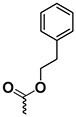	HCl	250	**125**	**62**.**5**	**125**	250	250	250
8		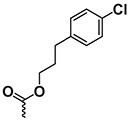	HCl	62.5	62.5	**<15**.**6**	31.25	62.5	62.5	31.25
9		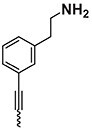	TFA	>125	125	**62**.**5**	**31.25**	**62.5**	125	>125
10		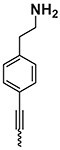	TFA	>125	>125	**62**.**5**	**62.5**	125	>125	>125
BDM91892 11		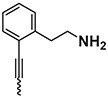	TFA	>62			**10**			
12		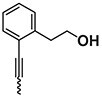		>62			> 62			

Bold values indicate boosting by at least 4-fold. AZM, azithromycin; CHL, chloramphenicol; CIP, ciprofloxacin; ERY, erythromycin; Fus, fusidic acid; HCl, hydrochloric acid; Nov, novobiocin; TFA, trifluoroacetic acid.

^a^Analogues 5, 11 and 12 were synthesized to probe structure-activity relationships of these compounds. CHL: 50 mg/L; Fus: 50 mg/L; Nov: 5 mg/L; ERY: 2.5 mg/L; AZM: 0.15 mg/L; and CIP: 0.2 mg/L.

A common characteristic among the most potent boosters, BDM91531 (compound 1) and BDM91892 (compound 11), is that they share a primary amine moiety. Replacing this group with another hydrogen bond-donating group, such as an alcohol, led to inactive compounds (compound 5, EC_90_ ≥ 125 µM; compound 12, EC_90_ > 62 µM), suggesting that the basic amine could interact with one of the acidic residues of the binding pocket via a salt bridge. In *E. coli*, both BDM91531 and BDM91892 were validated to inhibit AcrB (EC_90_ = 0.24 and 1.5 µM, respectively) as PyrPip resistance mutations (S446P or A450P substitution in AcrB)^13^ prevented antibiotic boosting activity of both compounds (EC_90_ = 32 µM) (Table S3).

### PyrPips inhibit A. baumannii RND pumps heterologously expressed in E. coli

Because *A. baumannii* expresses multiple RND efflux pumps, we studied the antibiotic boosting activity of PyrPips BDM91531 (compound 1) and BDM91892 (compound 11) on individual *A. baumannii* RND pumps using a heterologous expression system in *E. coli* lacking AcrAB. The *adeABC*, *adeFGH* or *adeIJK* operons were cloned into the *E. coli* Δ*acrAB* chromosome under the native promoter of *acrA*, to mediate a natural level of protein expression to PyrPip inhibition (pump overexpression could reduce EPI potency due to target overexpression). Quantitative PCR (qPCR) analyses confirmed that all three pumps were expressed at a comparable level in the recombinant strains (Table S4) although we recognize that this does not guarantee the correct and or expected assembly of the tripartite pumps in the *E. coli* membranes. Nonetheless, whereas expression of *adeABC* or *adeFGH* had no obvious impact on the growth rate, the *adeIJK* recombinant strain showed a moderate growth defect (Figure S1), similar to that previously reported.^5,27,28^

Initially, the impact of Ade efflux pump expression in *E. coli* Δ*acrAB* was determined by susceptibility to a selected panel of antibiotics (Table 2). Overall, heterologous expression of *adeABC* had limited impact on antibiotic susceptibility, which may be due to inefficiencies in AdeABC pump assembly in *E. coli* or to intrinsic differences in *E. coli* and *A. baumannii* cell wall architecture preventing proper functioning of this specific RND pump. This lack of AdeABC-related phenotype prevented the analysis of PyrPip inhibition on this RND pump. On the other hand, expression of *adeFGH* increased resistance to chloramphenicol and oxacillin (as well as the detergent SDS), whereas expression of *adeIJK* increased resistance to fusidic acid, novobiocin and erythromycin. These antibiotic profiles are largely in agreement with previous studies where these pumps were overexpressed, though the magnitude of resistance differs probably due to differences in expression level.^29,30^

**Table 2.** Antibiotic susceptibility (MIC in mg/L) for efflux-deficient *E. coli* Δ*acrAB* and *E. coli* Δ*acrAB* recombinant strains expressing *A. baumannii* RND efflux pumps AdeABC, AdeFGH or AdeIJK from the chromosomal *acrA* promoter

	*E. coli* Δ*acrAB*				*E. coli* Δ*acrAB:adeABC*				*E. coli* Δ*acrAB:adeFGH*				*E. coli* Δ*acrAB:adeIJK*			
	No EPI	91531	91892	PAβN	No EPI	91531	91892	PAβN	No EPI	91531	91892	PAβN	No EPI	91531	91892	PAβN
Chloramphenicol	2	2	1	2	2	1	2	1.7	**8**	1	2	2	1	1	2	2
Oxacillin	2	2	1	1	2	1	1	1	**32**	8	7	1	1	1	1	1
Piperacillin	0.5	0.5	0.5	0.5	0.5	0.5	0.5	0.5	0.5	0.5	0.5	0.5	0.5	0.5	0.5	0.5
Fusidic acid	8	3	5	0.5	8	1	8	5.3	13	2	2	3.3	**21**	3	2	0.5
Novobiocin	4	2	4	1	7	5	5	4	4	2	4	2	**16**	3	3	2
Linezolid	8	5	7	5	8	7	13	13	8	4	8	19	13	7	11	19
Erythromycin	5	3	3	0.5	8	2	2	0.5	8	2	2	0.5	**10.7**	3	2	0.7
Ciprofloxacin	0.005	0.005	0.005	0.005	**0.01**	0.005	0.005	0.005	**0.01**	0.005	0.005	0.005	**0.01**	0.005	0.005	0.005
SDS (%)	0.01	0.01	0.01	0.01					**0.06**	0.001	0.001	0.001	**0.04**	0.002	0.002	0.001

EPIs were added at a final concentration of 40 µM. Bold numbers show when Ade pump expression increased antibiotic resistance at least 2-fold. Antibiotic susceptibility was measured in liquid culture by resazurin microtiter assay (REMA). Data depict the mean MIC of at least three separate biological replicates. 91531 = BDM91531, 91892 = BDM91892.

Next, we challenged *E. coli* Δ*acrAB* and recombinant strains expressing *A. baumannii* RND efflux pumps in the presence of 40 µM BDM91531, BDM91892 or PAβN (Table 2). Results showed that all three molecules were able to increase antibiotic susceptibility. In particular, chloramphenicol and oxacillin resistance—mediated by *adeFGH* expression—was affected. Moreover, resistance to fusidic acid, novobiocin and erythromycin—mediated by *adeIJK* expression—was also reduced. In all cases, PyrPips were also able to decrease SDS resistance mediated by *adeFGH* and *adeIJK* expression. As *adeABC* expression did not give a clear resistance phenotype, it was not possible to evaluate the efficacy of the EPIs on this pump. Together, these data strongly support that both BDM91531 and BDM91892 can inhibit the activity of AdeFGH and AdeIJK when expressed in *E. coli*.

### Spectra of BDM91531 and BDM91892 activity on A. baumannii antibiotic susceptibility

**Figure 2.**
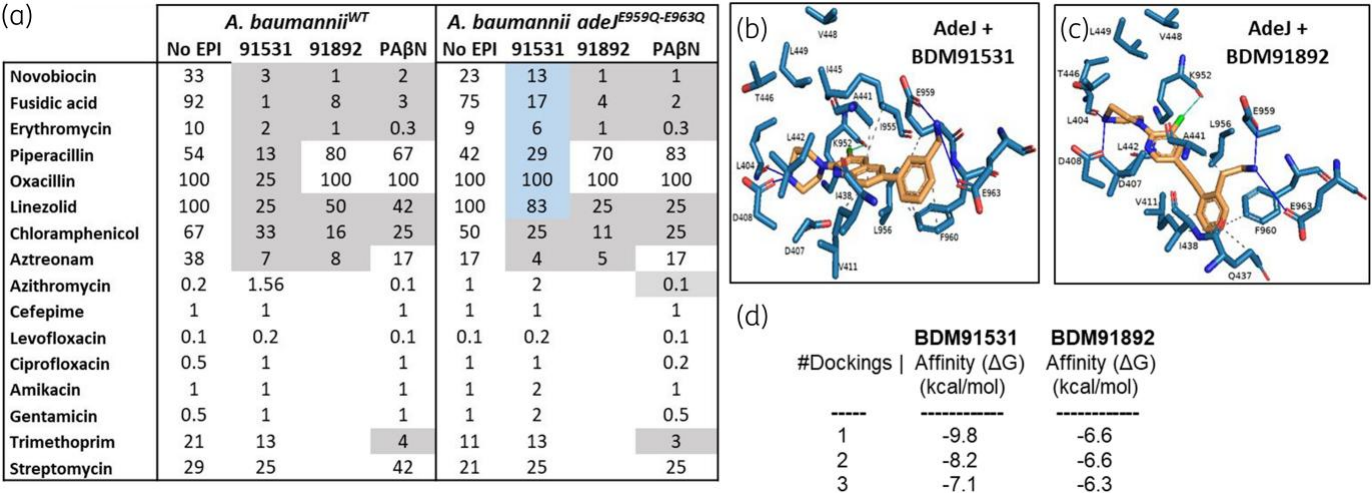
(a) MIC (mg/L) for a panel of antibiotics in the presence or absence of 40 µM EPI (BDM91531, BDM91892 or PAβN) against *A. baumannii* (WT) and isogenic strain with AdeJ E959Q-E963Q substitution. Conditions where EPIs decreased antibiotic MIC are indicated in grey-shaded cells (≥2-fold). Conditions where EPIs were less efficient at antibiotic boosting in the adeJ mutant compared with in the WT are highlighted in blue. Values are the mean MICs of at least three biological replicates. (b and c) Structural modelling highlighting protein–ligand interactions of the AdeJ efflux pump with either BDM91531 or BDM91892. Dotted lines: hydrophobic interactions; green line: halogen interactions; blue lines: polar interactions. (d) Free energy values for ligand affinity.

To characterize the spectra of antibiotic boosting by BDM91531 and BDM91892 in *A. baumannii* beyond those used in the PyrPips’ screening, we increased the antibiotics panel to 16 and included PAβN as an EPI control. Results largely reproduced the antibiotic boosting observed during the PyrPips’ screening (i.e. chloramphenicol, novobiocin, erythromycin and fusidic acid), but also showed boosting of linezolid, aztreonam and trimethoprim (and piperacillin and oxacillin by BDM91531 only); however, no boosting was observed for levofloxacin, amikacin, gentamicin, cefepime and streptomycin by any of the inhibitors (Figure 2). When comparing these data with observations from the heterologous expression system in *E. coli* Δ*acrAB*, it suggests that the observed antibiotic boosting in *A. baumannii* by BDM91531, BDM91892 or PAβN is through AdeIJK inhibition.

### Target confirmation of PyrPips in A. baumannii

Because *A. baumannii* encodes at least three RND pumps that are described to impact directly antibiotic susceptibility—though they are not equally expressed—we evaluated the relative expression of *adeB*, *adeG* and *adeJ* by qPCR. Data showed that *A. baumannii* primarily expresses *adeIJK* [average cycle threshold (Ct) = 19], followed by *adeABC* and *adeFGH* at much lower levels (average Ct = 24.35 and 26.40, respectively) (Table S4). Although transcriptional analysis does not fully reflect protein production levels, results are in line with previous data establishing AdeIJK as the predominant RND pump in *A. baumannii* strains.^5^

Structure-activity relationship data on PyrPips showed the primary amine moiety of BDM91531 and BDM91892 to be beneficial for potency. In fact, recent studies on *E. coli* AcrB showed that two acidic residues at the entrance of the PyrPip binding pocket in AcrB (E947 and D951) are crucial for PyrPip activity.^14^ Indeed, substitutions of any of these residues in AcrB led to a shift in potency for compounds BDM91531 and BDM91892, whereas PAβN activity was unaffected (Table S3). *A. baumannii* AdeJ also contains acidic residues at the equivalent positions (E959 and E963) (Figure 1). In the absence of an Ade-PyrPip co-structure or molecular dynamics data, *in silico* docking studies of BDM91531 and BDM91892 into the modelled AdeJ were deployed to evaluate PyrPip binding. The results showed that both EPIs could fit into the PyrPip binding pocket of AdeJ while making potential polar interactions between the PyrPip primary amine and the AdeJ acidic residues, E959 and E963. In addition to conservation of interactions with TM4 and TM5, a partial perpendicular π-stacking was also observed with AdeJ residue F960. Free energy (Δ*G*) calculations for each EPI suggested BDM91531 to have greater binding affinity to AdeJ compared with BDM91892 ($\Delta G^{\circ }$ = −9.8 and −6.6 kcal/mol, respectively; Figure 2d).

**Figure 3.**
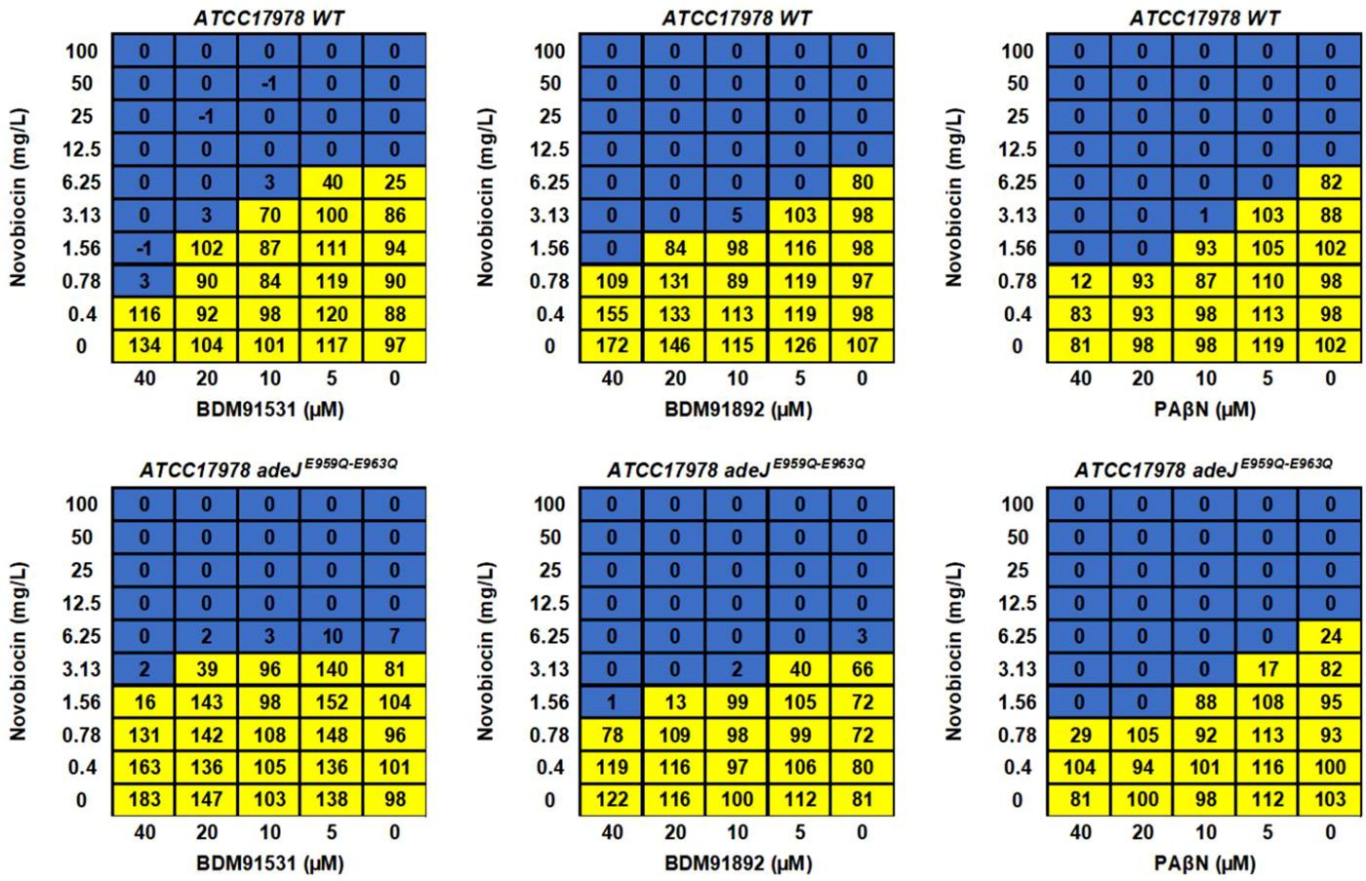
Checkerboard assay showing the synergy between novobiocin activity and the EPIs: BDM91531, BDM91892 or PAβN anti-*A. baumannii*. Susceptibility studies were performed on *A. baumannii* ATCC17978 WT strain and isogenic strain with AdeJ E959Q-E963Q substitution. Values indicate the bacterial viability expressed as the mean percentage of resazurin reduction assay (yellow cells >10%, blue cells <10%). Data represent the median bacterial viability of at least three biological replicates.

To probe the above predicted protein–ligand interaction accuracy, isogenic mutants of *A. baumannii* producing AdeJ with either E959Q, E963Q or E959Q-E963Q substitutions were generated. Antibiotic susceptibility testing showed that *A. baumannii* AdeJ^E959Q-E963Q^ was more resistant to the antibiotic-boosting impact of BDM91531 (particularly with novobiocin and fusidic acid) but not to BDM91892 or PAβN (all at 40 µM) (Figure 2a). In addition, checkerboard assays were used to further determine synergy between EPI and novobiocin (Figure 3) or fusidic acid (Figure S2). Our data were corroboration that compared with *A. baumannii* WT strain, the AdeJ^E959Q-E963Q^ mutant was more resistant to BDM91531-mediated boosting for novobiocin (Figure 2) and partially resistant to the boosting for fusidic acid (Figure S2). In contrast, BDM91892 and PAβN remained equally active (Figures S2 and 3). The observation that BDM91892 remained active in the mutant strain suggests that the AdeJ E959 and E963 residues do not play a major role in governing its activity, which agrees with its lower docking score (Figure 2d and Figure S2). As a contol, checkerboard assays were performed with *A. baumannii* lacking AdeJ (Δ*adeJ*) further confirming the role of this pump in novobiocin and fusidic acid efflux (Figure S2) and, thus, supporting that AdeJ is a primary target of PyrPips.

## Discussion

PyrPips have been demonstrated to be effective EPIs in *E. coli*, targeting an allosteric pocket in the TM region of AcrB.^13,14^ Based on the similarity of the residues lining the PyrPip binding pocket between *E. coli* AcrB and *A. baumannii* AdeB, AdeG and AdeJ pumps, we tested PyrPips for boosting antibiotic activity in *A. baumannii*. The combined results reveal that some PyrPips are indeed capable of inhibiting *A. baumannii* RND efflux pumps, particularly AdeG and AdeJ, as shown in the heterologous expression system in *E. coli*. Moreover, our data also show that in *A. baumannii* ATCC17978 AdeJ was the likely target responsible for the antibiotic-boosting phenotype. In particular, BDM91531 was able to boost novobiocin activity in *A. baumannii* by interacting with AdeJ acidic residues E959 and E963 in the PyrPip binding pocket. Overall, the data indicate that PyrPips’ activity is significantly less potent in *A. baumannii* than in *E. coli*, and that the observed structure-activity relationships of these molecules is not fully conserved. Likely contributing factors are differences in PyrPip penetration into *A. baumannii*, RND efflux pump expression levels, and the unstudied contribution of amino acid variations between AcrB and Ade pumps.

The main aim of this work was to establish a rational foundation towards a deeper understanding of how to improve and implement PyrPip EPIs against the current clinically relevant *A. baumannii* bacterium. We present systematic and strong evidence that PyrPip molecules can inhibit *A. baumannii* RND efflux pumps and that their co-exposure with antibiotics allows for synergistic antibiotic activity. Future efforts in unveiling the full mechanism of action behind PyrPips in *A. baumannii* will aim to develop more active inhibitors. Therefore, our current basic work provides an attractive strategy—currently in line with the WHO general agenda—to increase R&D to mitigate antimicrobial resistance driven by efflux pumps.

## Supplementary Material

dlad112_Supplementary_Data

## Supplementary data

Figures S1 and S2, Tables S1 to S4, and Supplementary Material are available as Supplementary data at *JAC-AMR* Online.
