# Supplementary material for: Characterization of pyridylpiperazine-based efflux pump inhibitors for Acinetobacter baumannii
Source: JAC Antimicrob Resist. 2023 Oct 24;5(5):dlad112. doi: 10.1093/jacamr/dlad112 (PMC10594211; doi:10.1093/jacamr/dlad112)
Supplement: dlad112_Supplementary_Data [file dlad112_supplementary_data.docx]

**Supplementary information**

**Characterisation of Pyridylpiperazine-based Efflux Pumps Inhibitors for *Acinetobacter baumannii***

Juan-Carlos Jiménez-Castellanos^1#^, Elizabeth Pradel^1#^, Nina Compagne^2^, Anais Vieira Da Cruz^2^, Marion Flipo^2^, Ruben C. Hartkoorn^1*^

# Joint first authors: Juan-Carlos Jiménez-Castellanos & Elizabeth Pradel. These authors contributed equally.

* Corresponding author: Ruben C Hartkoorn. E. mail: [ruben.hartkoorn@inserm.fr](mailto:ruben.hartkoorn@inserm.fr)

Affiliations:

1 Univ. Lille, CNRS, Inserm, CHU Lille, Institut Pasteur de Lille, U1019 - UMR 9017 - CIIL - Center for Infection and Immunity of Lille, F-59000 Lille, France.

2 Univ. Lille, Inserm, Institut Pasteur de Lille, U1177 - Drugs and Molecules for Living Systems, F-59000, Lille, France.

Running Title: PyrPip efflux pump inhibitors for *A. baumannii*

**Table S1: Bacterial strains and plasmids used in this work**

| **BACTERIAL STRAINS** | | |
| --- | --- | --- |
| **Strain Name** | **Characteristics** | **Source** |
| ***Escherichia coli*** | | |
| BW25113 | Keio collection from *E. coli* Genetic Stock Centre (CGSC, New Haven, Connecticut) | Baba, 2006 ^1^ |
| AcrB-A446P | BW25113 acrB-A446P mutant | Plé, 2019 ^2^ |
| AcrB-S450P | BW25113 acrB-S450P mutant | Plé, 2019 ^2^ |
| AcrB-E947Q | BW25113 acrB-E947Q mutant | Compagne, 2023 ^3^ |
| AcrB-D951Q | BW25113 acrB-D951N mutant | Compagne, 2023 ^3^ |
| EP511 | BW25113 Δ*acrAB* | this work |
| UCC pir+ | pir+ strain, able to replicate oriR6K plasmids | Lucigen, UK |
| JC0 | BW25113 Δ*acrAB* with pJC0 chromosomal integration at the *acrR* locus, KmR | this work |
| JC1 | BW25113 Δ*acrAB*::*adeIJK* due to pJC1 chromosomal integration at the *acrR* locus, KmR | this work |
| JC2 | BW25113 Δ*acrAB*::*adeABC* due to pJC2 chromosomal integration at the *acrR* locus, KmR | this work |
| JC3 | BW25113 Δ*acrAB*::*adeFGH* due to pJC3 chromosomal integration at the *acrR* locus, KmR | this work |
| EP1563 | BW25113 Δ*acrAB*::Gn, derived from JC0 | this work |
| EP1564 | BW25113 Δ*acrAB*::*adeABC*::Gn, derived from JC2 | this work |
| EP1565 | BW25113 Δ*acrAB*::*adeFG*H::Gn, derived from JC3 | this work |
| EP1566 | BW25113 Δ*acrA*B::*adeIJK*::Gn, derived from JC1 | this work |
|  |  |  |
| ***Acinetobacter baumannii*** | | |
| AB5075 | WT strain | Manoil Lab (Seattle, WA, USA) |
| ATCC 17978 | WT strain | BCCM/LMG, Gent, Belgium |
| ABEP3 | ATCC17978 Δ*adeJ* | this work |
| ABEP16 | ATCC17978 adeJ-E959Q-E963Q | this work |
|  |  |  |
| **PLASMIDS** | | |
| pCR-BluntII-TOPO | cloning vector, KmR | Thermo |
| pEP669 | source of GnR cassette | E. Pradel |
| pEP850 | oriR6K mobRP4 suicide plasmid, KmR | E. Pradel |
| pEP1436 | λ-Red-recombination plasmid, oriTs, AmpR | Valderrama, 2019 ^4^ |
| pKD4 | oriR6K, source of KmR cassette | Datsenko, 2000 ^5^ |
| pFLP2 | FLP recombinase expressed at 37°C, *sacB*, AmpR | Hoang, 1998 ^6^ |
| pJC0 | oriR6K mobRP4 bearing *acrR* and Pacr, KmR | this work |
| pJC1 | oriR6K mobRP4 bearing *acrR* and Pacr::adeIJK, KmR | this work |
| pJC2 | oriR6K mobRP4 bearing *acrR* and Pacr::adeABC, KmR | this work |
| pJC3 | oriR6K mobRP4 bearing *acrR* and Pacr::adeFGH, KmR | this work |
| pMo130 | non replicative in *A. baumannii*, *sacB*, KmR | Amin, 2013 ^7^ |
| pEP1529 | pMo130 bearing *adeJ* flanking regions, KmR | this work |
| pEP1571 | pCR-bluntII-TOPO bearing '*adeJK*' WT fragment, KmR | this work |
| pEP1574 | derived from pEP1571 after SDM on *adeJ*, to generate AdeJ-E963Q, KmR | this work |
| pEP1575 | derived from pEP1574 after 2nd *adeJ* SDM to generate AdeJ-E959Q-E963Q, KmR | this work |
| pEP1582 | pMo130 bearing 'adeJK' with mutations in *adeJ* to generate AdeJ-E959Q-E963Q, KmR | this work |

**Table S2: Primers used**

| **Primer Name** | **Sequence (5’-3’)** | **Use** |
| --- | --- | --- |
| RH717b | ATGAACAAAAACAGAGGGTTTACGCCTCTGGCGGTCGTTCTGATGGTGTAGGCTGGAGCTGCTTC | *acrAB* deletion construction |
| RH718b | TCAATGATGATCGACAGTATGGCTGTGCTCGATATCTTCATTCTTCATATGAATATCCTCCTTAG | *acrAB* deletion construction |
| RH719b | TGTATGTACCATAGCACGACGA | *acrAB* deletion check |
| RH720b | TGAGATCCTGAGTTGGTGGT | *acrAB* deletion check |
| RH886 | ACCTCGAGGATCCCGGGTAC**AGAATATCACGACGCAGTGAAC** | acrR-Pacr amplification and assembly |
| RH887 | GCTTTTGCAT**ATGTAAACCTCGAGTGTCCG** | acrR-Pacr amplification and assembly |
| RH888 | AGGTTTA**CATATGCAAAAGCATCTTTTACTTC** | adeABC amplification and assembly under Pacr into pEP850 |
| RH893 | TTATTTGATGCCTCTAGAGC**TTAGACTTTTGATATTCCTCCTCC** | adeABC amplification and assembly under Pacr into pEP850 |
| RH895 | AAAATGA**CATATGTAAACCTCGAGTGTCCG** | used with RH886 to assemble acrR-Pacr upstream of adeFGH |
| RH896 | AGGTTTA**CATATGTCATTTTCCCGCAAACAG** | adeFGH amplification and assembly under Pacr into pEP850 |
| RH901 | TTATTTGATGCCTCTAGAGC**TTAACTACTCCAACCGCCCC** | adeFGH amplification and assembly under Pacr into pEP850 |
| RH903 | CCGACAT**CATATGTAAACCTCGAGTGTCCG** | used with RH886 to assemble acrR-Pacr upstream of adeIJK |
| RH904 | AGGTTTACAT**ATGATGTCGGCTAAGCTTTG** | adeIJK amplification and assembly under Pacr into pEP850 |
| RH909 | TTATTTGATGCCTCTAGAGC**TTATTGCTTTTTAAGTTCAGC** | adeIJK amplification and assembly under Pacr into pEP850 |
| RH921 | TCCCGGGTACCAGAATATCA | adeIJK sequencing |
| RH922 | ATGATGTCGGCTAAGCTTTG | adeIJK sequencing |
| RH923 | TGACCCGTTGGTAACGATTC | adeIJK sequencing |
| RH924 | ATTGCACCACCAACCGTAAC | adeIJK sequencing |
| RH925 | TCGCGTAGAATTAGGTTCGG | adeIJK sequencing |
| RH926 | TGTAGCGTTGACGTTCACAC | adeIJK sequencing |
| RH927 | TGTAGATCAGGCTCAAGCTG | adeIJK sequencing |
| RH928 | GATTATTGCGGTTATCGGTC | adeIJK sequencing |
| RH929 | ATTTCTGGGGTCGTGTTCGT | adeIJK sequencing |
| RH930 | AGCACAACAACGTCTAGTAG | adeIJK sequencing |
| RH933 | TCCCGGGTACAGAATATCAC | adeABC sequencing |
| RH934 | ATGTGTTGGCGCGTTTCTTG | adeABC sequencing |
| RH935 | TATGAGCGCTTACAAGCTGC | adeABC sequencing |
| RH936 | AGCAGAGATTACCGCTACGT | adeABC sequencing |
| RH937 | CCGAAGGTGTTCGAGCAAAA | adeABC sequencing |
| RH938 | TGCCAGAGGAAGATCAAGGT | adeABC sequencing |
| RH939 | ACGCCACAATGGAATAAGGC | adeABC sequencing |
| RH940 | ATCGGCAATCGTATCTCGTG | adeABC sequencing |
| RH941 | GTCCAGACCTTAAAGCTGCC | adeABC sequencing |
| RH942 | TCCCGGGTACAGAATATCAC | adeFGH sequencing |
| RH943 | ATGTGTTGGCGCGTTTCTTG | adeFGH sequencing |
| RH944 | TCTGTATCACGCCTATACGC | adeFGH sequencing |
| RH945 | AGCGACGGTAACCTAACCAT | adeFGH sequencing |
| RH946 | TATGACCCGACTCAGTTCGT | adeFGH sequencing |
| RH947 | TCGTACCGAAGCAGTCATTC | adeFGH sequencing |
| RH948 | TTGCTGCACAAACTCTACCG | adeFGH sequencing |
| RH949 | ACTGGAAGATTGCCCAACCT | adeFGH sequencing |
| RH950 | ACGTATTGGGATTGCTCGTG | adeFGH sequencing |
| RH978 | CCAGTTTGTCCTGAGCAGAA | to check recombinant plasmid integration into Ec acrR locus |
| RH996 | TTATTTGATGCCTCTAGAGC**ATGTAAACCTCGAGTGTC** | used with RH886 to assemble acrR-Pacr into pEP850 |
| RH1241 | AATGGTAGAGGCCAACCT | rpoD qRTPCR-F |
| RH1242 | ACCAAGTCGCATAAGTCG | rpoD qRTPCR-R |
| RH1243 | TCGCTTACCGCAAGTTGTAC | adeB qRTPCR-F |
| RH1244 | CCTACACCTTCGACACGTTT | adeB qRTPCR-R |
| RH1245 | TACAAGGGGTTGGTGAAGTC | adeG qRTPCR-F |
| RH1246 | CCGCTGCAACCTGAATGTTT | adeG qRTPCR-R |
| RH1247 | TGAACAAGGTGTTGACCCTG | adeJ qRTPCR-F |
| RH1248 | CAAGAAGCTCGCACCAGATT | adeJ qRTPCR-R |
| RH1282b | CCCTAGACCTAGGCACAAATTGTTATCCGCTCACAATTCCACATGCGAATCCATGTGGGAGTTTA | to amplify GnR for recombineering |
| RH1283 | TCAATGATGATCGACAGTATGGCTGTGCTCGATATCTTCATTCTTTTAGGTGGCGGTACTTGGGT | to amplify GnR for recombineering |
| RH1357 | AGCTCATAAGCAGTTACACCCA | used with RH927 to clone 'adeJK' for site directed mutagenesis |
| RH1358 | GTCTTTCTGCAAAAAATGCGATCTTGATTGTTCAGTTCGCAAAACAGTTGCAGGAAAAAGGTGA | adeJ-E959Q-E963Q site directed mutagenesis |
| RH1359 | TCACCTTTTTCCTGCAACTGTTTTGCGAACTGAACAATCAAGATCGCATTTTTTGCAGAAAGAC | adeJ-E959Q-E963Q site directed mutagenesis |
| RH1360 | CACCTTTTTCCTGCAACTGTTTTGCGAATTCAACAATCAAGATCGCATT | adeJ-E963Q site directed mutagenesis |
| RH1361 | AATGCGATCTTGATTGTTGAATTCGCAAAACAGTTGCAGGAAAAAGGTG | adeJ-E963Q site directed mutagenesis |

**Table S3: Antibiotic boosting by EPI in *E. coli*'s AcrB variants.** Data show the effective concentration of EPI needed to cause 90% inhibition (EC_90_) of resazurin turnover (viability surrogate) of *E. coli* BW25113 WT, and variants AcrB-A446P, AcrB-S450P, AcrB-E947Q, and AcrB-D951N when grown in the presence of 8 mg/L pyridomycin (sub MIC concentration of AcrAB-TolC substrate antibiotic). Data are mean values of at least two biological replicates.

|  | ***E. coli* WT** | ***E. coli* A446P** | ***E. coli* S450P** | ***E. coli* E947Q** | ***E. coli* D951N** |
| --- | --- | --- | --- | --- | --- |
| **BDM88855.HCl** | 4 | >250 | >250 | 12 | 16 |
| **BDM91531** | 0.24 | 32 | 32 | 8 | 12 |
| **BDM91892** | 1.5 | 32 | 32 | 24 | 16 |
| **PAβN** | 16 | 16 | 16 | 16 | 12 |

**Table S4:** RND-efflux pump expression in **A**) *A. baumannii* WT and **B**) *E. coli* recombinant strains. Data shows the relative expression of the inner-membrane transporter component (*adeB, G* and ade*J*) relative to that of the respective house keeping gene (*rpoD*). Data were measured by QPCR and are expressed as mean ± SD of the difference in Ct values. QPCR experiments were performed in three independent biological replicates, with three technical replicates each. ND = not detected.

| **A** | **Expression level compared to *A.baumannii* *rpoD* (ΔCt)** | | |
| --- | --- | --- | --- |
|  | ***adeB*** | ***adeG*** | ***adeJ*** |
| ***A. baumannii*_WT** | -6.01 ± 1.19 | -6.92 ± 0.29 | 1.02 ± 0.84 |

| **B** | **Expression level compared to *E. coli ΔacrAB rpoD* (ΔCt)** | | |
| --- | --- | --- | --- |
|  | ***adeB*** | ***adeG*** | ***adeJ*** |
| ***E. coli*_adeB** | 3.84 ± 0.13 | ND | ND |
| ***E. coli*_adeG** | ND | 1.96 ± 0.19 | ND |
| ***E. coli*_adeJ** | ND | ND | 2.57 ± 0.19 |

**Figure S1: Growth curves of *E. coli* Δ*acrAB* and recombinant strains harbouring *A. baumannii* RND-efflux pump operons.** Data are the mean ± SEM of at least three independent biological replicates.

**Figure S2: Checkerboard assay evaluating novobiocin and fusidic acid antibiotic activity on *A. baumanni* strains in the presence of different concentrations of BDM91531, BDM91892, or PAβN.** Susceptibility studies were performed on *A. baumannii* (WT), isogenic mutant containing a double mutation in *adeJ* (E959Q-E963Q), and *ΔadeJ*. Values indicate the bacterial viability expressed as the mean percentage of resazurin reduction assay (yellow cells >10 %, blue cells <10%). Data represent the median bacterial viability of at least three biological replicates.

**Chemical synthesis of compounds 1-12**

*6-bromo-3-chloro-1H-quinolin-2-one (****1a****)*

To a solution of 6-bromo-1H-quinolin-2-one (22.3 mmol, 1.0 eq.) in anhydrous DMF (50.0 mL) was added NCS (1.3 eq.) and the reaction was stirred at 60 °C overnight. The DMF was evaporated and the compound was rinsed with water, EtOAc and MeOH and filtered under vacuum to give **1a** as a pink solid.

**Yield**: 73%; **LCMS (ES+)** *m/z* [M+H]^+^ 258; **^1^H NMR (300 MHz, CD_2_Cl_2_):** δ 7.28 (d, *J* = 8.8 Hz, 1H), 7.67 (dd, *J* = 2.3, 8.8 Hz, 1H), 7.92 (d, *J* = 2.2 Hz, 1H), 8.27 (s, 1H), 12.42 (s, 1H) ppm.

*6-bromo-2,3-dichloro-quinoline (****1b****)*

To a solution of 6-bromo-3-chloro-1H-quinolin-2-one **1a** (16.3 mmol, 1.0 eq.) in POCl_3_ (28 eq.). The mixture was stirred at 100 °C for 1h, then reaction mixture was poured on ice, basified with a saturated solution of Na_2_CO_3_ until pH= 7. The aqueous solution was extracted thrice with AcOEt. The organic layer was washed with brine, dried over MgSO_4_ and then evaporated under reduced pressure to give **1b** as a brown solid.

**Yield**: 73%; **LCMS (ES+)** *m/z* [M+H]^+^ 276; **^1^H NMR (300 MHz, CD_2_Cl_2_):** δ 7.82 (dd, *J* = 2.0, 9.0 Hz, 1H), 7.87 (d, *J* = 8.9 Hz, 1H), 7.97 (d, *J* = 1.4 Hz, 1H), 8.20 (s, 1H, ^4^CH) ppm.

*tert-butyl 4-(6-bromo-3-chloro-2-quinolyl)piperazine-1-carboxylate (****1c****)*

6-bromo-2,3-dichloro-quinoline **1b** (10.7 mmol, 1.0 eq.), piperazine (2.2 eq.), NEt_3_ (2.2 eq.) were dissolved in MeCN (39.0 mL) under argon. The mixture was heated at 80 °C for 2 days, cooled to room temperature. The reaction was washed with a 1N HCl aqueous solution, extracted twice with EtOAc. The organic layers were combined, washed with a saturated solution of NaCl, dried over MgSO_4_, evaporated under vacuum and purified by flash chromatography with a gradient of cyclohexane/EtOAc 100:0 to 90:10 to give **1c** as a pale yellow solid.

**Yield**: 85%; **LCMS (ES+)** *m/z* [M+H]^+^ 426; **^1^H NMR (300 MHz, CD_2_Cl_2_):** δ 1.47 (s, 9H), 3.41-3.45 (m, 4H), 3.58-3.62 (m, 4H), 7.67-7.68 (m, 2H), 7.80-7.81 (m, 1H), 7.99 (s, 1H) ppm.

General protocol for the synthesis of compounds **1d**, **3a** and **5a**:

The *tert*-butyl 4-(6-bromo-3-chloro-2-quinolyl)piperazine-1-carboxylate **1c** (0.2 mmol, 1.0 eq), appropriate phenylboronic acid (1.0 eq) and K_2_CO_3_ (1.6 eq) were dissolved in DME/EtOH/H_2_O (2/1/2, 5.0 mL) under argon. Then was added Pd(PPh_3_)_2_Cl_2_ (0.09-0.1 eq.), the suspension was heated at 90 °C under argon for 1h to 5h. The reaction was quenched with water, extracted twice with CH_2_Cl_2_. The organic layer was washed with brine, dried over MgSO_4_, evaporated under reduced pressure and purified by flash chromatography.

*tert-butyl 4-[6-[3-[(tert-butoxycarbonylamino)methyl]phenyl]-3-chloro-2-quinolyl]piperazine-1-carboxylate (****1d****)*

Purification flash chromatography (cyclohexane/EtOAc 100/0-70/30) to give the title compound as a yellow oil. **Yield**: 55%; **LCMS (ES+)** m/z 553 (MH^+^); **^1^H NMR** (300 MHz, CD_2_Cl_2_): δ 1.46 (s, 9H), 1.48 (s, 9H), 3.43-3.47 (m, 4H), 3.60-3.64 (m, 4H), 4.38 (d, *J* = 9.3 Hz, 2H), 5.04 (br s, 1H), 7.28-7.32 (m, 1H), 7.42-7.48 (m, 1H), 7.58-7.62 (m, 2H), 7.85-7.89 (m, 3H), 8.13 (s, 1H) ppm.

*tert-butyl 4-[6-[4-[(tert-butoxycarbonylamino)methyl]phenyl]-3-chloro-2-quinolyl]piperazine-1-carboxylate (****3a****)*

Purification by flash chromatography (cyclohexane/EtOAc 100/0-80/20) to give the title compound as a white solid; **Yield**: 55%; **LCMS (ES+)** m/z 553 (MH^+^); **^1^H NMR** (300 MHz, CD_2_Cl_2_): δ 1.47 (s, 9H), 1.49 (s, 9H), 3.43-3.46 (m, 4H), 3.61-3.64 (m, 4H), 4.35 (d, *J* = 6.1 Hz, 2H), 5.13 (br s, 1H), 7.39 (d, *J* = 8.2 Hz, 2H), 7.65 (d, *J* *=* 8.2 Hz, 2H), 7.83-7.87 (m, 3H), 8.10 (s, 1H) ppm.

*tert-butyl 4-[3-chloro-6-[3-(hydroxymethyl)phenyl]-2-quinolyl]piperazine-1-carboxylate (****5a****)*

Purification by flash chromatography (cyclohexane/EtOAc 100/0-60/40) to give the title compound as a colourless oil; **Yield**: 29%; **LCMS (ES+)** m/z 454 (MH^+^); **^1^H NMR** (300 MHz, CD_2_Cl_2_): δ 1.48 (s, 9H), 1.91 (t, 1H), 3.43-3.47 (m, 4H), 3.61-3.64 (m, 4H), 4.77 (d, *J* = 5.7 Hz, 2H), 7.38 (d, *J* = 7.7 Hz, 1H), 7.48 (t, *J* = 7.6 Hz, 1H), 7.62 (td, *J* = 1.6, 8.0 Hz, 1H), 7.70-7.72 (m, 1H), 7.86-7.93 (m, 3H), 8.13 (s, 1H) ppm.

General protocol for the synthesis of compounds **1**, **3** and **5**:

In a round-bottomed flask containing the corresponding Boc-protected compound (0.07-0.13 mmol, 1.0 eq.) in 1,4-dioxane (0.4-0.6 mL) was added HCl 4M in 1,4-dioxane (20-21 eq.). The mixture was stirred at room temperature overnight. The solvent was then evaporated under reduced pressure, petroleum ether was added and the mixture was filtered to give the desired compounds.

*[3-(3-chloro-2-piperazin-1-yl-6-quinolyl)phenyl]methanamine;dihydrochloride (****1****)*

Yellow solid, **Yield**: 91%; **^1^H NMR** (300 MHz, DMSO-*d*_6_): δ 3.25-3.30 (m, 4H), 3.65-3.70 (m, 4H), 4.11 (q, *J* = 5.6 Hz, 2H), 7.53-7.58 (m, 2H), 7.77-7.82 (m, 1H), 7.91 (d, *J* = 8.8 Hz, 1H), 8.02 (s, 1H), 8.09 (dd, *J* = 2.1, 8.8 Hz, 1H), 8.24 (d, *J* = 1.9 Hz, 1H), 8.50 (s, 1H), 8.64 (br s, 3H), 9.65 (br s, 2H) ppm; **^13^C NMR** (75 MHz, DMSO-*d*_6_): δ 42.2, 42.4, 45.8, 122.0, 124.4, 125.9, 126.7, 127.7, 127.7, 128.3, 129.1, 129.3, 135.0,  136.5, 138.3, 139.3, 144.1, 155.7 ppm; **HRMS (*m/z*)**: [M+H]^+^ calcd. for C_20_H_22_ClN_4_ 353.1533; found 353.1525

*[4-(3-chloro-2-piperazin-1-yl-6-quinolyl)phenyl]methanamine;dihydrochloride (****3****)*

White solid, **Yield**: 85%; **^1^H NMR** (300 MHz, DMSO-*d*_6_): δ 3.26-3.30 (m, 4H), 3.64-3.69 (m, 4H), 4.08 (s, 2H), 7.64 (d, *J =* 8.4 Hz*,* 2H), 7.84 (d, *J =* 8.3 Hz, 2H), 7.89 (d, *J =* 8.8 Hz, 1H), 8.06 (dd, *J =* 2.1, 8.8 Hz, 1H), 8.22 (d, *J =* 2.0 Hz*,* 1H), 8.52 (s, 1H), 8.58 (s, 2H), 9.56 (s, 3H) ppm; **^13^C NMR** (75 MHz, DMSO-*d*_6_): δ 41.8, 42.5, 45.8, 121.9, 124.4, 126.0, 126.9, 127.7, 129.1, 129.7, 133.6, 136.3, 138.3, 139.2, 144.1, 155.6 ppm; **HRMS (*m/z*)**: [M+H]^+^ calcd. for C_20_H_22_ClN_4_ 353.1533; found 353.1530.

*[3-(3-chloro-2-piperazin-1-yl-6-quinolyl)phenyl]methanol;hydrochloride (****5****)*

Yellow solid, **Yield**: 100%; **^1^H NMR** (300 MHz, DMSO-*d*_6_): δ 3.26-3.32 (m, 4H), 3.63-3.68 (m, 4H), 4.60 (s, 2H), 7.36 (d, *J =* 7.6 Hz*,* 1H), 7.48 (t, *J =* 7.6 Hz*,* 1H), 7.64 (d, *J =* 7.8 Hz*,* 1H), 7.74 (s, 1H), 7.89 (d, *J =* 8.8 Hz*,* 1H), 8.03 (dd, *J =* 2.1, 8.8 Hz, 1H), 8.18 (d, *J =* 2.0 Hz*,* 1H), 8.55 (s, 1H), 9.46 (br s, 2H) ppm; **^13^C NMR** (75 MHz, DMSO-*d*_6_): δ 42.5, 45.8, 62.8, 121.4, 124.2, 124.9, 125.2, 125.9, 126.0, 127.6, 128.9,  129.2, 137.2, 138.4, 138.9, 143.5, 144.0, 155.5 ppm; **HRMS (*m/z*)**: [M+H]^+^ calcd. for C_20_H_21_ClON_3_ 354.1373; found 354.1392

*tert-butyl 4-[3-chloro-6-(4,4,5,5-tetramethyl-1,3,2-dioxaborolan-2-yl)-2-quinolyl]piperazine-1-carboxylate (****2a****)*

In a round-bottomed flask was added tert-butyl 4-(6-bromo-3-chloro-2-quinolyl)piperazine-1-carboxylate **1c** (2.34 mmol, 1.0 eq.), bis(pinacolato)diboron (2.52 mmol, 1.1 eq.), K_2_CO_3_ (7.37 mmol, 3.1 eq.), Pd(dppf)Cl_2_ (0.07 mmol, 0.03 eq.), dissolved in 1,4-dioxane (50 mL). The mixture was degassed for 40 minutes then heated to 100°C. After one night, Bis(pinacolato)diboron (0.41 mmol, 0.2 eq.), K_2_CO_3_ (2.25 mmol, 1.1 eq.) and Pd(dppf)Cl_2_ (0.02 mmol, 0.01 eq.) were added then the mixture was stirred for 3h. The reaction mixture was then washed with water and then extracted thrice with dichloromethane. The organic layers were washed with a saturated NaCl solution, dried over MgSO_4_ and evaporated under vacuum. The crude product was purified by flash chromatography (cyclohexane/EtOAc 100/0-90/10) to give the title compound **2a**.

**Yield**: 73%; **LCMS (ES+)** m/z 474 (MH^+^); **^1^H NMR** (300 MHz, CD_2_Cl_2_): δ 1.36 (s, 12H), 1.47 (s, 9H), 3.44-3.47 (m, 4H), 3.59-3.62 (m, 4H), 7.76 (d, *J* = 8.4 Hz, 1H), 7.93 (dd, *J* = 1.4, 8.4 Hz, 1H), 8.08 (s, 1H), 8.11 (br s, 1H) ppm.

*tert-butyl N-[(2-bromophenyl)methyl]carbamate (****2b****)*

LiAlH_4_ (1 M in THF, 2.0 eq.) was dissolved in THF (5.0 mL) then was added dropwise a solution of AlCl_3_ (2.0 eq.) in THF/ Et_2_O (5:1, 6.0 mL). The solution was stirred at room temperature for 20 min. Then 2-bromobenzonitrile (2.8 mmol, 1.0 eq.) in THF (4.0 mL) was added dropwise. The suspension was stirred at room temperature for 3h, then was quenched with water at 0 °C and stirred at room temperature for 1h. The reaction was quenched with NH_3_ in H_2_O, extracted twice with CH_2_Cl_2_. The organic layer was washed with brine, dried over MgSO_4_ and evaporated under reduced pressure to afford to (2-bromophenyl)methanamine. The crude product (1.42 mmol, 1.0 eq.) was dissolved in CH_2_Cl_2_ (2.5 mL) then a solution of di-*tert*-butyl dicarbonate (1.3 eq.) in CH_2_Cl_2_ (2.5 mL) was added dropwise at 0°C. The reaction was stirred at room temperature for 1h. The crude product was evaporated under reduced pressure to tert-butyl N-[(2-bromophenyl)methyl]carbamate **2b** as a yellow oil.

**Yield**: 62% over 2 steps; **LCMS (ES+)** m/z 286 (MH^+^); **^1^H NMR** (300 MHz, CD_2_Cl_2_): δ 1.42 (s, 9H), 4.35 (d, *J* = 6.2 Hz, 2H), 5.08 (br s, 1H), 7.12-7.19 (m, 1H), 7.31 (td, *J* = 1.2, 7.6 Hz, 1H), 7.35-7.39 (m, 1H), 7.54 (dd, *J* = 1.1, 7.9 Hz, 1H) ppm.

*tert-butyl 4-[6-[2-[(tert-butoxycarbonylamino)methyl]phenyl]-3-chloro-2-quinolyl]piperazine-1-carboxylate (****2c****)*

The tert-butyl 4-[3-chloro-6-(4,4,5,5-tetramethyl-1,3,2-dioxaborolan-2-yl)-2-quinolyl]piperazine-1-carboxylate **2a** (0.3 mmol, 1.0 eq), tert-butyl *N*-[(2-bromophenyl)methyl]carbamate **2b** (1.1 eq) and K_2_CO_3_ (1.7 eq) were dissolved in DME/EtOH/H_2_O (2/1/2, 5.0 mL) under argon. Then was added Pd(PPh_3_)_2_Cl_2_ (0.1 eq.), the suspension was heated at 90 °C under argon for 3h. The reaction was quenched with water, extracted twice with CH_2_Cl_2_. The organic layer was washed with brine, dried over MgSO_4_, evaporated under reduced pressure. The crude product was purified by flash chromatography (cyclohexane/EtOAc 100/0-80/20) to give the title compound **2c**.

**Yield:** 68%; **LCMS (ES+)** m/z 553 (MH^+^); **^1^H NMR** (300 MHz, CD_2_Cl_2_): δ 1.43 (s, 9H), 1.48 (s, 9H), 3.43-3.47 (m, 4H), 3.61-3.65 (m, 4H), 4.26 (d, *J* = 5.9 Hz, 2H), 4.82 (br s, 1H), 7.29-7.48 (m*,* 4H), 7.56-7.60 (m*,* 2H), 7.85 (dd, *J* = 0.6, 9.2 Hz, 1H), 8.09 (s, 1H) ppm.

*[2-(3-chloro-2-piperazin-1-yl-6-quinolyl)phenyl]methanamine;dihydrochloride (****2****)*

In a round-bottomed flask containing *tert*-butyl 4-[6-[2-[(*tert*-butoxycarbonylamino)methyl]phenyl]-3-chloro-2-quinolyl]piperazine-1-carboxylate **2c** (0.20 mmol, 1.0 eq.) in 1,4-dioxane (1.6 mL) was added HCl 4M in 1,4-dioxane (20 eq.). The mixture was stirred at room temperature overnight. The solvent was then evaporated under reduced pressure, petroleum ether was added and the mixture was filtered to give the title compound **2** as yellow solid.

**Yield**: 99%; **^1^H NMR** (300 MHz, DMSO-*d*_6_): δ 3.26-3.32 (m, 4H), 3.66-3.70 (m, 4H), 3.93-3.98 (m, 2H), 7.37-7.41 (m, 1H), 7.46-7.56 (m, 2H), 7.72-7.80 (m, 2H), 7.89 (d, *J* = 8.7 Hz, 1H), 7.93 (d, *J* = 1.7 Hz, 1H), 8.53 (s, 1H), 8.63 (br s, 3H), 9.61 (br s, 2H) ppm; **^13^C NMR** (75 MHz, DMSO-*d*_6_): δ 39.2, 42.4, 45.9, 122.0, 125.5, 127.1, 127.3, 128.1, 128.4, 128.9, 130.3, 131.5, 131.6, 136.6, 138.3, 140.6, 143.8, 155.9 ppm; **HRMS (*m/z*)**: [M+H]^+^ calcd. for C_20_H_22_ClN_4_ 353.1533; found 353.1540

*tert-butyl 4-[6-[4-(tert-butoxycarbonylamino)butylamino]-3-chloro-2-quinolyl]piperazine-1-carboxylate (****4a****)*

*tert*-butyl 4-(6-bromo-3-chloro-2-quinolyl)piperazine-1-carboxylate **1c** (0.23 mmol, 1.0 eq.), *tert*-butyl *N*-(4-aminobutyl)carbamate (0.35 mmol, 1.5 eq.), Cs_2_CO_3_ (0.34 mmol, 1.5 eq.), Xantphos (0.02 mmol, 0.08 eq.), Pd_2_dba_3_ (0.01 mmol, 0.04 eq.) were dissolved in 1,4-dioxane (1.4 mL) under argon. The mixture was heated at 100 °C overnight, cooled to room temperature, filtered on celite, dried under vacuum. The crude product was purified by flash chromatography (cyclohexane/EtOAc 100/0-80/20) to give the title compound **4a** as yellow oil.

**Yield**: 18%; **LCMS (ES+)** m/z 534 (MH^+^); **^1^H NMR** (300 MHz, CD_2_Cl_2_): δ 1.42 (s*,* 9H), 1.47 (s*,* 9H), 1.59-1.73 (m, 4H), 3.11-3.23 (m, 4H), 3.26-3.30 (m, 4H), 3.57-3.61 (m, 4H), 4.04 (br s, 1H), 4.64 (br s, 1H), 6.59 (d, *J =* 2.6 Hz, 1H), 7.02 (dd, *J =* 2.6*,* 9.0 Hz*,*1H), 7.59 (d, *J =* 9.0 Hz, 1H), 7.88 (s, 1H) ppm.

*N'-(3-chloro-2-piperazin-1-yl-6-quinolyl)butane-1,4-diamine;dihydrochloride (****4****)*

In a round-bottomed flask containing tert-butyl 4-[6-[4-(*tert*-butoxycarbonylamino)butylamino]-3-chloro-2-quinolyl]piperazine-1-carboxylate **4a** (0.04 mmol, 1.0 eq.) in 1,4-dioxane (0.4 mL) was added HCl 4M in 1,4-dioxane (19 eq.). The mixture was stirred at room temperature overnight. The solvent was then evaporated under reduced pressure, petroleum ether was added and the mixture was filtered to give the title compound **4** as brown solid.

**Yield**: 96%; **^1^H NMR** (300 MHz, DMSO-*d*_6_): δ 1.67-1.72 (m, 4H), 2.74-2.83 (m, 2H), 3.15-3.28 (m, 6H), 3.49-3.53 (m, 4H), 6.93 (s, 1H), 7.31 (dd, *J =* 2.6, 9.0 Hz*,* 1H), 7.63 (d, *J =* 9.0 Hz*,* 1H), 8.04 (br s, 3H), 8.24 (s, 1H), 9.45 (br s, 2H) ppm; **^13^C NMR** (75 MHz, DMSO-*d*_6_): δ 24.7, 38.4, 42.6, 43.5, 46.2, 121.9, 122.4, 127.5, 127.9, 136.0 ppm**; HRMS (*m/z*)**: [M+H]^+^ calcd. for C_17_H_25_ClN_5_ 334.1798; found 334.1795

*tert-butyl 4-(3-chloro-5-methoxycarbonyl-2-pyridyl)piperazine-1-carboxylate (****6a****)*

In a tube were introduced the corresponding methyl 5,6-dichloropyridine-3-carboxylate (4.1 mmol, 1 eq.), *tert*-butyl piperazine-1-carboxylate (1.5 eq.) and triethylamine (1.3 eq.) in 8 mL of acetonitrile. The resulting mixture was stirred at 80 °C during 16 hours. The solvent was evaporated and the residue dissolved in EtOAc. The organic layer was washed with aqueous HCl 1N. The aqueous layer was then washed three times with EtOAc. The combined organic layers were washed with brine, dried over MgSO_4_, and evaporated under reduced pressure. The crude was purified by flash chromatography (cyclohexane/EtOAc 100:0 to 80:20) to give **6a** as a light orange powder.

**Yield:** quantitative; **LC-MS (ESI)** m/z [M+H]^+^ = 356; **^1^H NMR** (300 MHz, CD_2_Cl_2_), δ (ppm): 1.46 (s, 9H), 3.46-3.51 (m, 4H), 3.52-3.57 (m, 4H), 3.87 (s, 3H), 8.13 (d, *J* = 1.9 Hz, 1H), 8.71 (d, *J* = 1.9 Hz, 1H).

*6-(4-tert-butoxycarbonylpiperazin-1-yl)-5-chloro-pyridine-3-carboxylic acid (****6b****)*

Compound **6a** (4.86 mmol, 1 eq.), NaOH (8 eq.) and 1.5 mL water were stirred in 20 mL MeOH at 65 °C for 1 h. The reaction was diluted with water and then acidified with an aqueous solution of HCl 1N. The aqueous layer was extracted thrice with EtOAc. The combined organic layers were washed with brine, dried over MgSO_4_ and evaporated under reduced pressure to give compound **6b** as a white solid.

**Yield:** quantitative; **LC-MS (ESI)** m/z [M+H]^+^ = 342; **^1^H NMR** (300 MHz, DMSO-*d_6_*), δ (ppm): 1.41 (s, 9H), 3.39-3.50 (m, 8H), 8.09 (d, *J* = 2.0 Hz, 1H), 8.66 (d, *J* = 2.0 Hz, 1H), 13.19 (s, 1H).

*tert-butyl 4-[5-[4-[2-(tert-butoxycarbonylamino)ethyl]phenoxy]carbonyl-3-chloro-2-pyridyl]piperazine-1-carboxylate (****6c****).*

Compound **6b** (0.50 mmol, 1 eq.) was stirred at r.t. with K_2_CO_3_ (2.5 eq) and COMU (1.5 eq.) for 10 min in EtOAc (1.5 mL). Then, *tert*-butyl *N*-[2-(4-hydroxyphenyl)ethyl]carbamate (1.5 eq.) was added and the reaction was allowed to stir at room temperature for 16h. The reaction was washed once with an aqueous solution of HCl 1N, twice with a saturated solution of NaHCO_3_ and the product was extracted with EtOAc. The combined organic layers were washed with brine, dried over MgSO_4_ and evaporated under vacuum. The intermediate was purified by flash chromatography (cyclohexane/EtOAc 100:0 to 70:30) to give compound **6c** as a colourless oil.

**Yield:** 46%; **LC-MS (ESI)** m/z [M+H]^+^ = 561; **^1^H NMR** (300 MHz, CD_2_Cl_2_), δ (ppm): 1.42 (s, 9H), 1.47 (s, 9H), 2.81 (t, *J* = 7.0 Hz, 2H), 3.36 (q, *J* = 6.8 Hz, 2H), 3.57 (s, 8H), 4.64 (brs, 1H), 7.11-7.16 (m, 2H), 7.24-7.29 (m, 2H), 8.26 (d, *J* = 2.1 Hz, 1H), 8.87 (d, *J* = 2.0 Hz, 1H).

*[4-(2-aminoethyl)phenyl] 5-chloro-6-piperazin-1-yl-pyridine-3-carboxylate;dihydrochloride (****6****).*

In a round-bottomed flask containing the compound **6c** (0.21 mmol, 1 eq.) in 1.0 mL of 1,4-dioxane, HCl 4M in 1,4-dioxane (10 eq.) was added. The mixture was stirred at room temperature during 16 hours. The solvent was then evaporated under reduced pressure, petroleum ether was added and the mixture was filtered to give **6** as a white powder.

**Yield:** 97%; **LC-MS (ESI)** m/z [M+H]^+^ = 361; **^1^H NMR** (300 MHz, DMSO-*d_6_*), δ (ppm): 2.89-3.11 (m, 4H), 3.17-3.28 (m, 4H), 3.77 (t, *J* = 4.8 Hz, 4H), 7.25 (d, *J* = 8.5 Hz, 2H), 7.36 (d, *J* = 8.6 Hz, 2H), 8.13-8.20 (m, 3H), 8.32 (d, *J* = 2.1 Hz, 1H), 8.87 (d, *J* = 2.0 Hz, 1H), 9.59 (brs, 2H); **^13^C NMR** (75 MHz, DMSO-*d_6_*), δ (ppm): 32.3, 40.3, 42.4, 45.0, 119.3, 119.6, 122.0, 129.8, 135.4, 140.0, 147.9, 149.1, 159.3, 162.6; **HRMS (TOF, ES+) m/z [M+H]^+^:** calcd. for C_18_H_22_ClN_4_O_2_ 361.1431, found 361.1422.

*2-phenylethyl 5-chloro-6-piperazin-1-yl-pyridine-3-carboxylate;hydrochloride (****7****).*

A 1 M solution of isopropyl chloroformate in toluene (1.5 eq.) was added in 2.5 mL of anhydrous THF under argon. Carboxylic acid **6b** (2.45 mmol, 1 eq.) and TEA (1.2 eq.) were dissolved in 2.5 mL of anhydrous THF and added dropwise to the isopropyl chloroformate solution at 0 °C. The reaction was allowed to warm up to r.t. and stirred overnight. A solution of saturated NaHCO_3_ was added and the product was extracted twice with EtOAc. The organic layer was washed once with a saturated solution of NaHCO_3_, once with brine, dried over MgSO_4_ and evaporated under reduced pressure.

A solution of the previous compound (0.5 mmol, 1 eq.) dissolved in 1 mL anhydrous THF was added dropwise at 0 °C to a solution of 2-phenylethanol (1.2 eq.) and *t*BuOK (1.6 eq.) dissolved in 1 mL anhydrous THF at 0 °C. The mixture was stirred at r.t. for 4h. The reaction was diluted with EtOAc and then washed twice with a 1N HCl aqueous solution and twice with a saturated solution of NaHCO_3_. The organic layers were combined, washed with a saturated solution of NaCl, dried over MgSO_4_ and evaporated under vacuum. The crude was purified by flash chromatography (cyclohexane/EtOAc 100:0 to 90:10). In a round-bottomed flask containing the obtained ester in 1.0 mL of 1,4-dioxane, HCl 4M in 1,4-dioxane (10 eq.) was added. The mixture was stirred at room temperature during 10 hours. The solvent was then evaporated under reduced pressure, petroleum ether was added and the mixture was filtered to give compound **7** as a white solid.

**Yield:** 37%; **LC-MS (ESI)** m/z [M+H]^+^ = 346; **^1^H NMR** (300 MHz, DMSO-*d_6_*), δ (ppm): 3.02 (t, *J* = 6.7 Hz, 2H), 3.19-3.22 (m, 4H), 3.69-3.72 (m, 4H), 4.47 (t, *J* = 6.6 Hz, 2H), 7.19-7.32 (m, 5H), 8.08 (d, *J* = 1.9 Hz, 1H), 8.64 (d, *J* = 1.9 Hz, 1H), 9.53 (brs, 2H); **^13^C NMR** (75 MHz, DMSO-*d_6_*), δ (ppm): 34.3, 42.4, 45.0, 65.5, 119.7, 120.1, 126.4, 128.4, 128.9, 138.0, 139.4, 147.1, 158.9, 163.5; **HRMS (TOF, ES+) m/z [M+H]^+^:** calcd. for C_18_H_21_ClN_3_O_2_ 346.1322, found 346.1328.

*tert-butyl 4-[3-chloro-5-[3-(4-chlorophenyl)propoxycarbonyl]-2-pyridyl]piperazine-1-carboxylate (****8a****)*

Compound **6b** (0.50 mmol, 1 eq.) was stirred at r.t. with TEA (2 eq.) and COMU (1.5 eq.) for 10 min in EtOAc (1.5 mL). Then, 3-(4-chlorophenyl)propan-1-ol (1.5 eq.) was added and the reaction was allowed to stir at room temperature for 16h. The reaction was washed once with an aqueous solution of HCl 1N, twice with a saturated solution of NaHCO_3_ and the product was extracted with EtOAc. The combined organic layers were washed with brine, dried over MgSO_4_ and evaporated under vacuum. The intermediate was purified by reverse phase chromatography (MeCN 0.1% formic acid/water 0.1% formic acid 10:90 to 100:0) to give compound **8a** as a colourless oil.

**Yield:** 55%; **LC-MS (ESI)** m/z [M+H]^+^ = 494; **^1^H NMR** (300 MHz, CD_2_Cl_2_), δ (ppm): 1.46 (s, 9H), 2.01-2.11 (m, 2H), 2.75 (t, *J* = 7.1 Hz, 2H), 3.47-3.58 (m, 8H), 4.29 (t, *J* = 6.6 Hz, 2H), 7.16 (d, *J* = 8.4 Hz, 2H), 7.26 (d, *J* = 8.4 Hz, 2H), 8.08 (d, *J* = 2.0 Hz, 1H), 8.70 (d, *J* = 2.0 Hz, 1H).

*3-(4-chlorophenyl)propyl 5-chloro-6-piperazin-1-yl-pyridine-3-carboxylate;hydrochloride (****8****)*

In a flask containing compound **8a** (0.26 mmol, 1 eq.) in 1 mL of dry 1,4-dioxane, HCl 4M (10 eq.) was added. The mixture was stirred at room temperature for 4h. The solvent was then evaporated under reduced pressure, petroleum ether was added and the mixture was filtered to give compound **8** as a light orange oil.

**Yield:** 97%; **LC-MS (ESI)** m/z [M+H]^+^ = 394; **^1^H NMR** (300 MHz, DMSO-*d_6_*), δ (ppm): 1.96-2.06 (m, 2H), 2.73 (t, *J* = 7.3 Hz, 2H), 3.19-3.25 (m, 4H), 3.70 (t, *J* = 4.6 Hz, 4H), 4.26 (t, *J* = 6.3 Hz, 2H), 7.25-7.34 (m, 4H), 8.08 (d, *J* = 2.0 Hz, 1H), 8.67 (d, *J* = 2.0 Hz, 1H), 9.36 (brs, 2H); **^13^C NMR** (75 MHz, DMSO-*d_6_*), δ (ppm): 29.5, 31.0, 42.5, 45.1, 64.4, 119.8, 120.2, 128.2, 130.3, 130.5, 139.4, 140.4, 147.2, 158.9, 163.6; **HRMS (TOF, ES+) m/z [M+H]^+^:** calcd. for C_19_H_22_Cl_2_N_3_O_2_ 394.1089, found 394.1095.

*tert-butyl 4-(5-bromo-3-chloro-2-pyridyl)piperazine-1-carboxylate* (**9a**)

2,3-dichloro-5-bromopyridine (8.8 mmol, 1 eq), *tert*-butyl piperazine-1-carboxylate  (3 eq.) and TEA (1.3 eq.) were stirred at 80°C in 12 mL MeCN for 7 days. The reaction was washed four times with an aqueous solution of HCl 1N and extracted twice with EtOAc. The organic layers were combined, washed with a saturated solution of NaCl, dried over MgSO_4_ and evaporated under reduced pressure. The crude was purified by flash chromatography with a gradient cyclohexane/EtOAc (100/0 to 90/10) to give the desired product **9a** as a colorless oil.

**Yield:** 82%; **LC-MS (ESI)** m/z [M+H]^+^ **=** 378; **^1^H NMR** (300 MHz, CD_2_Cl_2_), δ (ppm): 1.45 (s, 9H), 3.24-3.29 (m, 4H), 3.51-3.56 (m, 4H), 7.75 (d, *J* = 2.2 Hz, 1H), 8.21 (d, *J* = 2.2 Hz, 1H).

*tert-butyl 4-(3-chloro-5-iodo-2-pyridyl)piperazine-1-carboxylate* (**9b**)

A round bottom flask was charged with CuI (5 mol%), sodium iodide (2 eq.) and compound **9a** (4.0 mmol, 1 eq.). The flask was purged with argon for 30 min then dry dioxane (8.7 mL) and trans-*N*1,*N*2-dimethylcyclohexane-1,2-diaminedioxane (0.1 eq.) were added. The solution was heated at 110 °C under argon. 5 mol% CuI, 2 eq NaI and 0.1 eq trans-*N*1,*N*2-dimethylcyclohexane-1,2-diaminedioxane were added after 7, 11, 13 and 15 days. 5 mL dioxane were also added after 12 days of reaction. The reaction was stopped after 16 days in total. The reaction mixture was diluted with EtOAc and was washed twice with H_2_O. The organic layer was then washed with brine, dried over MgSO_4_ and evaporated under reduced pressure. The crude was purified by flash chromatography with a gradient cyclohexane/EtOAc (100/0-70/30) to give the title compound **9b** as a light yellow solid.

**Yield:** 77%; **LC-MS (ESI)** m/z [M+H]^+^ **=** 424; **^1^H NMR** (300 MHz, CD_2_Cl_2_), δ (ppm): 1.45 (s, 9H), 3.25-3.30 (m, 4H), 3.50-3.56 (m, 4H), 7.88 (d, *J* = 2.0 Hz, 1H), 8.33 (d, *J* = 2.0 Hz, 1H).

*tert-butyl 4-[3-chloro-5-(2-trimethylsilylethynyl)-2-pyridyl]piperazine-1-carboxylate* (**9c**)

In a tube, were added compound **9b** (1.65 mmol, 1 eq), Pd(PPh_3_)_2_Cl_2_ (2 mol%) and CuI (6 mol%). The tube was purged with argon for 15 min, then ethynyl(trimethyl)silane (1.5 eq), TEA (13 eq) and 6 mL anhydrous MeCN were added. The reaction was heated at 100°C under microwaves for 1h30. The reaction was filtered on a celite plug. The residue was purified by flash chromatography with a gradient of cyclohexane/EtOAc (100/0-90/10) to give the desired product **9c** as a light yellow solid.

**Yield:** 54%; **LC-MS (ESI)** m/z [M+H]^+^ **=** 394; **^1^H NMR** (300 MHz, CD_2_Cl_2_), δ (ppm): 0.23 (s, 9H), 1.45 (s, 9H), 3.29-3.38 (m, 4H), 3.49-3.58 (m, 4H), 7.64 (d, *J* = 2.0 Hz, 1H), 8.21 (d, *J* = 2.0 Hz, 1H).

*tert-butyl 4-(3-chloro-5-ethynyl-2-pyridyl)piperazine-1-carboxylate* (**9d**)

TBAF 1M in THF (1.1 eq) was added to a solution of compound **9c** (1.47 mmol, 1 eq) in 6 mL THF. The reaction was allowed to stir at RT for 3 h. The reaction was diluted with EtOAc and washed twice with water. The aqueous layer was extracted once with EtOAc. The combined organic layers were then washed with brine, dried over MgSO_4_ and evaporated under reduced pressure to give the desired product **9d** as a brown solid.

**Yield:** 93%; **LC-MS (ESI)** m/z [M+H]^+^ **=** 322; **^1^H NMR** (300 MHz, CD_2_Cl_2_), δ (ppm): 1.46 (s, 9H), 3.21 (s, 1H), 3.32-3.38 (m, 4H), 3.51-3.57 (m, 4H), 7.68 (d, *J* = 2.0 Hz, 1H), 8.26 (d, *J* = 2.0 Hz, 1H).

*2-(2-iodophenyl)ethanol* (**9e**)

To a solution of 2-(2-iodophenyl)acetic acid (1.00 mol, 1.0 eq) in 2.5 mL of dry THF was added (CH_3_)_2_S·BH_3_ 2M in THF (1.1 eq). The mixture was stirred 3.5 h at RT. The THF was evaporated. The residue was dissolved in EtOAc and the resulting solution was washed once with water, once with a saturated solution of NaHCO_3_ and once with brine. The organic layer was then dried over MgSO_4_ and evaporated under reduced pressure to give the desired alcohol **9e** as a colorless liquid.

**Yield:** 78%; **LC-MS (ESI)** m/z not ionizable; **^1^H NMR** (300 MHz, CD_2_Cl_2_), δ (ppm): 1.47 (t, *J* = 5.8 Hz, 1H), 2.99 (t, *J* = 6.8 Hz, 2H), 3.82 (q, *J* = 6.2 Hz, 2H), 6.86-6.96 (m, 1H), 7.25-7.34 (m, 2H), 7.82-7.87 (m, 1H).

General protocol Sonogashira coupling to synthesize compounds **9f**, **10a**, **11a** and **12a**:

In a tube, were added compound **9d** (0.23-0.62 mmol), Pd(PPh_3_)_2_Cl_2_ (2-3 mol%), CuI (6-10 mol%) and the corresponding iodo derivative (1.4 eq.). Then TEA (14 eq.) and 0.9-2.5 mL anhydrous MeCN were added. The reaction was flushed under argon for 30 min and was heated at 100°C under microwaves for 1 h. The reaction was filtered on a celite plug. The filtrate was diluted with EtOAc and was washed three times with water and once with brine. It was then dried over MgSO_4_, filtered and the solvent was removed under reduced pressure. The crude was purified by flash chromatography.

*tert-butyl 4-[5-[2-[3-[2-(tert-butoxycarbonylamino)ethyl]phenyl]ethynyl]-3-chloro-2-pyridyl]piperazine-1-carboxylate* (**9f**)

Purification by flash chromatography (cyclohexane/EtOAc 100/0-80/20); **Yield:** 52%; **LC-MS (ESI)** m/z [M+H]^+^ **=** 541; **^1^H NMR** (300 MHz, CD_2_Cl_2_), δ (ppm): 1.41 (s, 9H), 1.46 (s, 9H), 2.79 (t, *J* = 7.0 Hz, 2H), 3.31-3.40 (m, 6H), 3.52-3.59 (m, 4H), 4.60 (brs, 1H), 7.20 (td, *J* = 1.4, 7.5 Hz, 1H), 7.31 (t, *J* = 7.8 Hz, 1H), 7.36-7.40 (m, 2H), 7.73 (d, *J* = 2.0 Hz, 1H), 8.31 (d, *J* = 2.0 Hz, 1H).

*tert-butyl 4-[5-[2-[4-[2-(tert-butoxycarbonylamino)ethyl]phenyl]ethynyl]-3-chloro-2-pyridyl]piperazine-1-carboxylate* (**10a**)

Purification by flash chromatography (cyclohexane/EtOAc 100/0-80/20); **Yield:** 76%; **LC-MS (ESI)** m/z [M+H]^+^ **=** 541; **^1^H NMR** (300 MHz, CD_2_Cl_2_), δ (ppm): 1.41 (s, 9H), 1.46 (s, 9H), 2.80 (t, *J* = 7.1 Hz, 2H), 3.29-3.40 (m, 6H), 3.52-3.59 (m, 4H), 4.59 (brs, 1H), 7.21 (d, *J* = 8.2 Hz, 2H), 7.46 (d, *J* = 7.3 Hz, 2H), 7.73 (d, *J* = 2.0 Hz, 1H), 8.30 (d, *J* = 2.0 Hz, 1H).

*tert-butyl 4-[5-[2-[2-[2-(tert-butoxycarbonylamino)ethyl]phenyl]ethynyl]-3-chloro-2-pyridyl]piperazine-1-carboxylate* (**11a**)

Purification by reverse phase flash chromatography (water/MeCN 90/10-0/100); **Yield:** 56%; **LC-MS (ESI)** m/z [M+H]^+^ **=** 541; **^1^H NMR** (300 MHz, CD_2_Cl_2_), δ (ppm): 1.39 (s, 9H), 1.46 (s, 9H), 3.02 (t, *J* = 7.0 Hz, 2H), 3.33-3.46 (m, 6H), 3.53-3.58 (m, 4H), 4.68 (brs, 1H), 7.20-7.34 (m, 3H), 7.51 (dd, *J* = 1.4, 8.0 Hz, 1H), 7.85 (brs, 1H), 8.34 (d, *J* = 2.0 Hz, 1H).

*tert-butyl 4-[3-chloro-5-[2-[2-(2-hydroxyethyl)phenyl]ethynyl]-2-pyridyl]piperazine-1-carboxylate* (**12a**)

Purification by flash chromatography (cyclohexane/EtOAc 100/0-70/30); **Yield:** 60%; **LC-MS (ESI)** m/z [M+H]^+^ **=** 442; **^1^H NMR** (300 MHz, CD_2_Cl_2_), δ (ppm): 1.46 (s, 9H), 3.10 (t, *J* = 6.7 Hz, 2H), 3.33-3.41 (m, 4H), 3.52-3.59 (m, 4H), 3.92 (t, *J* = 6.6 Hz, 2H), 7.21-7.33 (m, 3H), 7.52 (td, *J* = 1.0, 7.4 Hz, 1H), 7.73 (d, *J* = 2.0 Hz, 1H), 8.32 (d, *J* = 2.0 Hz, 1H).

General protocol deprotection to synthesize compounds **9** to **12**:

In a flask containing the corresponding Boc-protected intermediate (0.18-0.32 mmol, 1 eq.) in 5.3-9.4 mL of dry DCM, TFA (40 eq) was added. The mixture was stirred at RT for 40 min to 2.5 h. The reaction was evaporated under reduced pressure and the oil rinsed several times with DCM. Diethyl ether was added to the obtained oil to make the product precipitate. It was filtered to give the desired product.

*2-[3-[2-(5-chloro-6-piperazin-1-yl-3-pyridyl)ethynyl]phenyl]ethanamine;2,2,2-trifluoroacetic acid* (**9**)

**Yield:** 78 %; **LC-MS (ESI)** m/z [M+H]^+^ **=** 341; **^1^H NMR** (300 MHz, DMSO-*d_6_*), δ (ppm): 2.89 (t, *J* = 7.5 Hz, 2H), 3.04-3.13 (m, 2H), 3.23-3.29 (m, 4H), 3.52-3.58 (m, 4H), 7.34 (td, *J* = 1.7, 7.2 Hz, 1H), 7.40 (d, *J* = 7.4 Hz, 1H), 7.44 (t, *J* = 1.8 Hz, 1H), 7.47 (d, *J* = 1.6 Hz, 1H), 7.94 (brs, 3H), 8.06 (d, *J* = 2.0 Hz, 1H), 8.43 (d, *J* = 2.0 Hz, 1H), 9.04 (brs, 2H) ppm; **^13^C NMR** (75 MHz, DMSO-*d*_6_), δ (ppm): 32.7, 39.7, 42.7, 45.5, 85.1, 91.9, 114.1, 120.8, 122.0, 129.2 , 129.7, 129.8, 131.6, 138.1, 141.1, 148.4, 156.0, 158.2 (q, *J* = 30.9 Hz, TFA); **HRMS (TOF, ES+) m/z [M+H]^+^:** calcd. for C_19_H_22_ClN_4_341.1533, found 341.1530.

*2-[4-[2-(5-chloro-6-piperazin-1-yl-3-pyridyl)ethynyl]phenyl]ethanamine;2,2,2-trifluoroacetic acid* (**10**)

**Yield:** 71%; **LC-MS (ESI)** m/z [M+H]^+^ **=** 341; **^1^H NMR** (300 MHz, DMSO-*d_6_*), δ (ppm): 2.87-2.92 (m, 2H), 3.01-3.14 (m, 2H), 3.21-3.30 (m, 4H), 3.51-3.59 (m, 4H), 7.34 (d, *J* = 8.3 Hz, 2H), 7.53 (d, *J* = 8.2 Hz, 2H), 7.87 (brs, 3H), 8.07 (d, *J* = 1.9 Hz, 1H), 8.43 (d, *J* = 1.9 Hz, 1H), 8.90 (brs, 2H); **^13^C NMR** (75 MHz, DMSO-*d_6_*), δ (ppm): 32.9, 39.6, 42.8, 45.5, 85.0, 91.9, 114.2, 120.2, 120.9, 129.3, 131.7, 138.6, 141.2, 148.4, 156.0; **HRMS (TOF, ES+) m/z [M+H]^+^:** calcd. for C_19_H_22_ClN_4_341.1533, found 341.1548.

*2-[2-[2-(5-chloro-6-piperazin-1-yl-3-pyridyl)ethynyl]phenyl]ethanamine;2,2,2-trifluoroacetic acid* (**11**)

**Yield:** 85%; **LC-MS (ESI)** m/z [M+H]^+^ **=** 341; **^1^H NMR** (300 MHz, DMSO-*d_6_*), δ (ppm): 3.11 (s, 4H), 3.21-3.33 (m, 4H), 3.53-3.61 (m, 4H), 7.31-7.46 (m, 3H), 7.56 (dd, *J* = 1.2, 7.5 Hz, 1H), 8.03 (brs, 3H), 8.12 (d, *J* = 2.0 Hz, 1H), 8.51 (d, *J* = 2.0 Hz, 1H), 9.08 (brs, 2H); **^13^C NMR** (75 MHz, DMSO-*d*_6_), δ (ppm): 32.0, 39.1, 42.7, 45.5, 89.0, 89.9, 114.1, 120.8, 121.6, 127.4, 129.5, 132.4, 139.0, 141.1, 148.6, 156.1, 158.3 (q, *J* = 30.4 Hz, TFA); **HRMS (TOF, ES+) m/z [M+H]^+^:** calcd. for C_19_H_22_ClN_4_341.1533, found 341.1521.

*2-[2-[2-(5-chloro-6-piperazin-1-yl-3-pyridyl)ethynyl]phenyl]ethanol* (**12**)

Purification by reverse phase chromatography (water 0.1% formic acid/MeCN 0.1% formic acid 10/90-0/100). **Yield:** quantitative; **LC-MS (ESI)** m/z [M+H]^+^ **=** 342; **^1^H NMR** (300 MHz, DMSO-*d_6_*), δ (ppm): 2.97 (t, *J* = 7.2 Hz, 2H), 3.06-3.17 (m, 4H), 3.40-3.52 (m, 4H), 3.66 (t, *J* = 7.2 Hz, 2H), 7.22-7.31 (m, 1H), 7.35 (d, *J* = 3.8 Hz, 2H), 7.50 (d, *J* = 7.4 Hz, 1H), 8.03 (d, *J* = 2.0 Hz, 1H), 8.43 (d, *J* = 2.0 Hz, 1H); **^13^C NMR** (75 MHz, DMSO-*d*_6_), δ (ppm): 38.1, 43.6, 46.6, 61.7, 88.7, 91.1, 114.6, 121.3, 121.9, 126.9, 129.6, 130.3, 132.3, 141.4, 141.5, 148.7, 156.6; **HRMS (TOF, ES+) m/z [M+H]^+^:** calcd. for C_19_H_21_ClN_3_O 342.1373, found 342.1371.

**References**

1. Baba T, Ara T, Hasegawa M, *et al.* Construction of Escherichia coli K-12 in-frame, single-gene knockout mutants: the Keio collection. *Mol Syst Biol* 2006; **2**: 2006.0008.

2. Plé C, Tam HK, Vieira Da Cruz A, *et al.* Pyridylpiperazine-based allosteric inhibitors of RND-type multidrug efflux pumps. *Nat Commun* 2022; **13**: 1–11.

3. Compagne N, Vieira Da Cruz A, Müller RT, Hartkoorn RC, Flipo M, Pos KM. Update on the Discovery of Efflux Pump Inhibitors against Critical Priority Gram-Negative Bacteria. *Antibiotics* 2023; **12**: 180.

4. Valderrama K, Pradel E, Firsov AM, *et al.* Pyrrolomycins Are Potent Natural Protonophores. *Antimicrob Agents Chemother* 2019; **63**: 1–15.

5. Datsenko KA, Wanner BL. One-step inactivation of chromosomal genes in Escherichia coli K-12 using PCR products. *Proc Natl Acad Sci U S A* 2000; **97**: 6640–5.

6. Hoang TT, Karkhoff-Schweizer RR, Kutchma AJ, Schweizer HP. A broad-host-range F1p-FRT recombination system for site-specific excision of chromosomally-located DNA sequences: Application for isolation of unmarked Pseudomonas aeruginosa mutants. *Gene* 1998; **212**: 77–86.

7. Amin IM, Richmond GE, Sen P, Koh TH, Piddock LJ, Chua KL. A Method for generating marker-less gene deletions in multidrug-resistant Acinetobacter baumannii. *BMC Microbiol* 2013; **13**.
